# Routine dyspnea assessment and documentation: Nurses’ experience yields wide acceptance

**DOI:** 10.1186/s12912-016-0196-9

**Published:** 2017-01-14

**Authors:** Kathy M. Baker, Susan DeSanto-Madeya, Robert B. Banzett

**Affiliations:** 1 Lois E. Silverman Department of Nursing, Beth Israel Deaconess Medical Center, 330 Brookline Avenue Reisman 1113, Boston, MA 02215 USA; 2 Connell School of Nursing, Boston College, Chestnut Hill, MA USA; 3 Department of Medicine, Division of Pulmonary, Critical Care, and Sleep Medicine, Beth Israel Deaconess Medical Center, Boston, MA USA; 4 Harvard Medical School, Boston, MA USA

**Keywords:** Dyspnea, Dyspnea assessment, Nurse assessment of dyspnea

## Abstract

**Background:**

Dyspnea (breathing discomfort) is a common and distressing symptom. Routine assessment and documentation can improve management and relieve suffering. A major barrier to routine dyspnea documentation is the concern that it will have a deleterious effect on nursing workflow and that it will not be readily accepted by nurses. Nurses at our institution recently began to assess and document dyspnea on all medical-surgical patients upon admission and once per shift throughout their hospitalization. A year after dyspnea measurement was implemented we explored nurses’ approach to dyspnea assessment, their perception of patient response, and their perception of the utility and burden of dyspnea measurement.

**Methods:**

We obtained feedback from nurses using a three-part assessment of practice: 1) a series of recorded focus group interviews with nurses, 2) a time-motion observation of nurses performing routine dyspnea and pain assessment, and 3) a randomized, anonymous on-line survey based, in part, on issues raised in focus groups.

**Results:**

Ninety-four percent of the nurses surveyed reported administering the dyspnea assessment is “easy” or “very easy”. None of the nurses reported that assessing dyspnea negatively impacted workflow and many reported that it positively improved their practice by increasing their awareness. Our time-motion data showed dyspnea assessment and documentation takes well less than a minute. Nurses endorsed the importance of routine measurement and agreed that most patients were able to provide a meaningful rating of their dyspnea. Nurses found the patient report very useful, and used it in conjunction with observed signs to respond to changes in a patient’s condition.

**Conclusions:**

In this study, we have demonstrated that routine dyspnea assessment and documentation was widely accepted by the nurses at our institution. Our nurses fully incorporated routine dyspnea assessment and documentation into their practice and felt that it improved patient-centered care.

**Electronic supplementary material:**

The online version of this article (doi:10.1186/s12912-016-0196-9) contains supplementary material, which is available to authorized users.

## Background

### Study aims

Dyspnea is a prevalent symptom in a wide variety of disease states, not limited to cardiopulmonary disorders. Dyspnea is often as distressing as the more commonly experienced symptom of pain. It has been argued that proper management of dyspnea, like management of pain, should be expected as standard of care [1–3]. Routine pain assessment and documentation is nearly universal; dyspnea assessment is not. Barriers to routine dyspnea documentation include concerns that it will have a deleterious effect on nursing workflow and that it will not be readily accepted by nurses [4]. The present study addresses these concerns.

Dyspnea assessment and documentation upon admission and once per shift throughout hospitalization for all medical-surgical patients was recently initiated at our institution. In this study, we explored nurses’ approach to dyspnea assessment, their perception of patient response, and their perception of the utility and burden of dyspnea measurement. Our assessment of practice included a time-motion study, focus group interviews, and a randomized anonymous on-line survey. All studies were approved by the Institutional Review Board (IRB) at Beth Israel Deaconess Medical Center (BIDMC). Our findings provide guidance for other institutions wishing to include dyspnea measurement in routine patient assessment.

### Routine dyspnea assessment in clinical practice

**Fig. 1:**
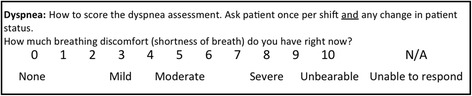
Dyspnea scale nurses use to record patient reported dyspnea. Anchor terms (None, Unbearable) are from the A_1_ scale of the Multidimensional Dyspnea Profile, a validated instrument [22]. Intermediate words added at the suggestion of nurses (Mild, Moderate, and Severe) are words frequently used for pain assessment, and placement was consistent with word-scaling data from pain patients and healthy persons [34]

Since April 2013, nurses on all medical-surgical units at our institution have been instructed to assess and document dyspnea on every patient at least once per nursing shift. Nurses used a single numeric scale on which zero was defined as ‘None’ and ten was defined as ‘Unbearable’ breathing discomfort (Fig. 1). These assessments were documented for the clinical record on paper flowsheets as part of a bundled assessment that also includes pain, fall risk, and agitation/sedation [5]. Before implementation, we taught nursing educators and clinical nurse specialists on all medical-surgical units the rationale for, and the approach to, routine assessment of dyspnea. The content was developed by authors RBB and KMB. (RBB has more than 25 years experience researching dyspnea mechanisms and dyspnea measurement; KMB is nurse educator with more than 32 years experience in nursing.) Unit educators received a longer form of the presentation and disseminated the information to clinical nurses via small group in-services or email copies of the PowerPoint program. In addition, our team delivered a shorter ‘inservice’ to a subset of nurses on every unit. We included these key points: dyspnea is a widespread and distressing symptom, it is often underestimated by nurses and physicians, dyspnea is what the patient says it is, and it can possibly predict adverse events in patients. We emphasized that the first step in managing this burdensome symptom is to measure it.

**Fig. 2:**
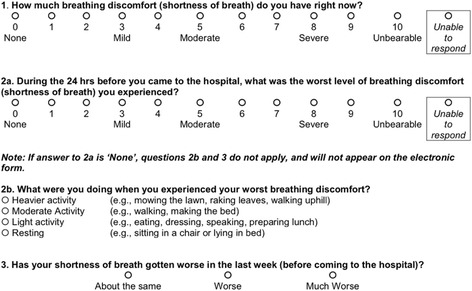
Nursing assessment of all medical surgical patients upon admission

Nurses complete an electronic Initial Patient Assessment (IPA) on all medical surgical patients within 8 h of admission. Since March 2014, the IPA includes the patient’s report of current dyspnea and recent history of dyspnea (Fig. 2). We previously reported our experience with the pilot version of this assessment [6].

## Methods

### Description of study components

#### Nursing focus groups

Between April and November 2014, a convenience sample of 63 nurses from six medical-surgical units participated in 12 half-hour focus group sessions. Eighty-two percent of the nurses who participated reported having received some educational training on dyspnea and the new dyspnea assessment scale. Focus session data were analyzed qualitatively using standard content analysis methods. Focus groups provided information on which to base a subsequent anonymous survey, as well as provided direct quotations from nurses to illustrate the issues.

A nurse researcher (SD-M) experienced with conducting focus groups asked for the nurses’ views on the following topics: the process nurses used for assessment of dyspnea, importance of dyspnea assessment and awareness, patients’ ability to rate and use the dyspnea scale, impact of routine assessment on workflow, and suggestions for improvement. The nurse researcher explained at the outset that the session was voluntary and that anonymity and confidentiality would be maintained. Research assistants and graduate student nurses attended the meetings and took notes; nurses were identified by number code only. Sessions were also recorded using an audio recorder to confirm verbatim quotations.

Focus group sessions were scheduled 2–3 days in advance in consultation with unit leadership and were held in unit conference rooms during regular work shifts. Nurses attending the focus groups were provided a fact sheet describing the purpose of the session, risks/benefits of participation, and reiteration that participation was entirely voluntary.

#### Time-motion study

An objective measure of the time nurses take to perform dyspnea assessment and documentation is key to understanding workload implications and to compare with nurses’ perceptions of the burden imposed. A total of 40 registered nurses representing the 14 medical-surgical inpatient units were chosen randomly for recruitment. Subjects were provided an information sheet describing the purpose of the study and verbal consent was obtained. All nurses who were asked to be observed for this study agreed to participate. The purpose of the observation was also explained to the patients. No protected patient information was recorded.

**Fig. 3:**
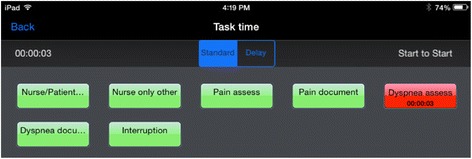
Screen shot of time motion app as it appears during the assessment of dyspnea by a nurse

The study took place between September 2014 and August 2015. Nurses were observed by a clinical nurse specialist (CNS) (KMB) familiar with hospital procedures who recorded the time nurses spent assessing and documenting pain and dyspnea. Data were recorded using a tablet (iPAD Mini) and time-motion application (TimeStudy, nuVizz, Atlanta GA; Fig. 3). The iPAD application allowed for easy pauses for interruptions in care. These observations occurred in inpatient rooms during the morning assessment of the patient by the nurse providing care. The CNS stood within 6 ft of the patient and used the touch screen to start and stop timing during the nurse-patient interaction.

#### Nursing survey

Additional feedback was obtained from the nurses using an anonymous on-line survey through REDCap [7]. The survey was sent to 70 nurses randomly selected from the 14 inpatient medical-surgical units, or approximately 10% of clinical nurses employed on those units. Names were selected randomly from the list of nurses on each unit, and the names were sent to nursing leaders on the respective units to ensure these nurses were appropriate candidates for the survey; nurses were not eligible for participation if they were on a medical leave, had been employed less than 6 months, or were per diem status and worked infrequent shifts. A replacement name was randomly selected for each nurse that did not meet eligibility criteria. Reminders to complete the survey were sent by nurse leaders at the institution.

The survey was sent in April 2015 and nurses were allowed 6 weeks to respond; 67 surveys (96%) were completed. Demographics can be found in the online supplement (Additional file 1: Table S1; Additional file 2: Table S2; Additional file 3: Table S3 and Additional file 4: Figure S1 & Additional file 5: Figure S2). The majority of responding nurses (73%) reported they had not attended a research focus group on dyspnea (Additional file 6: Figure S3). Most nurses (67%) reported receiving education on the new dyspnea assessment: of those receiving training, 78% received an informal in-service, 20% received a PowerPoint presentation with question and answer, and 40% received an email announcement (Additional file 7: Figure S4 & Additional file 8: Figure S5).

## Results

### Effect of routine dyspnea assessment and documentation on practice

#### Perceived importance of dyspnea assessment and documentation

**Fig. 4:**
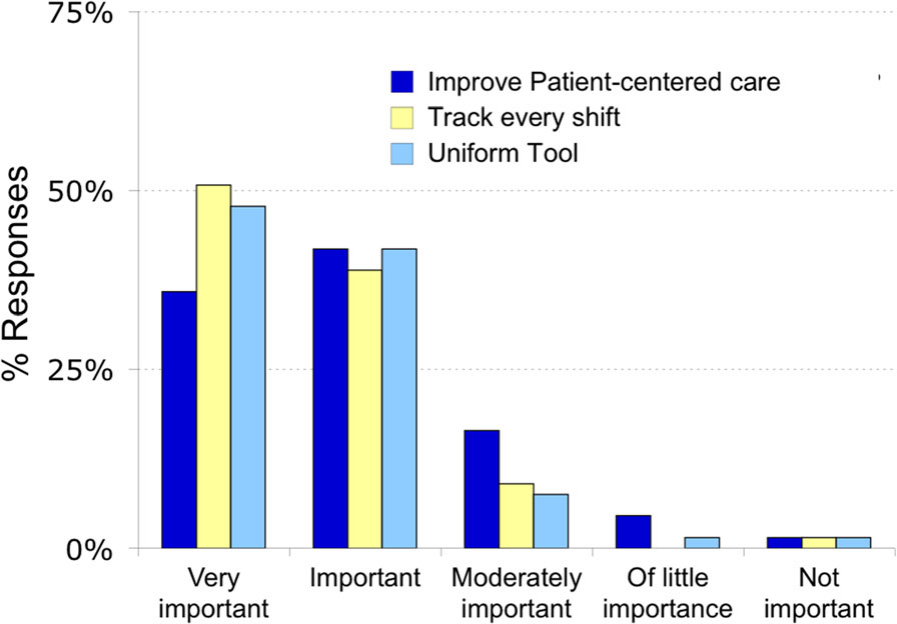
Nurses’ opinions of the importance of routine assessment

Nurses agreed that assessing and recording dyspnea in patients is important. The overwhelming majority felt it was ‘important’ or ‘very important’ to assess (94%) and document (97%) dyspnea upon admission, to use a uniform tool to assess for dyspnea (90%), and to track dyspnea every shift (90%). A strong majority of nurses responded that routine assessment of dyspnea is ‘important’ or ‘very important’ in improving patient centered care (78%) (Fig. 4, Additional file 9: Figures S6; Additional file 10: Figure S7; Additional file 11: Figure S8; Additional file 12: Figure S9; Additional file 13: Figure S10; Additional file 14: Figure S11). These results were exemplified by the following comments from the focus group sessions and were further supported by survey responses:

*“Yes, now we compare previous scores and see if there has been an improvement.”*

*“*[Assessing] *shortness of breath does assist the medical team to see….changes and help with the management and treatment of our patients.”*

*“…it can be the first sign of bad things to come.”*

Most importantly, the nurses reported that implementation of routine dyspnea assessment has had a positive impact on their nursing practice as demonstrated by the following comments recorded from the focus groups:

*“It’s not something I really focused on before, so I think the fact that the tool is there, reminds me to be more attentive.”*

*“You have patients that look like they are resting easy and you ask them about it* [dyspnea] *and they say, ‘I’ve felt short of breath for the last 2 days.’ I never would have asked about that before.”*

#### Impact of routine dyspnea assessment on workflow

Nurses overwhelmingly (94%) reported that performing routine dyspnea assessment is “easy” or “very easy” and many (42%) reported that the implementation of q-shift assessment had “positively” or “very positively” affected workflow (Additional file 15: Figure S12 & Additional file 16: Figure S13).

Nurses commented that dyspnea assessment was already a routine part of patient care, although it was not systematically documented. As two nurses explained during the focus groups:

*“I feel like it’s always been part of our workflow, I don’t think adding a number takes much more time.”*

*“It’s just like a motion. It’s part of my routine, now, because we have to document it. It’s part of what I just do, it doesn’t really hinder or make a difference.”*

**Fig. 5:**
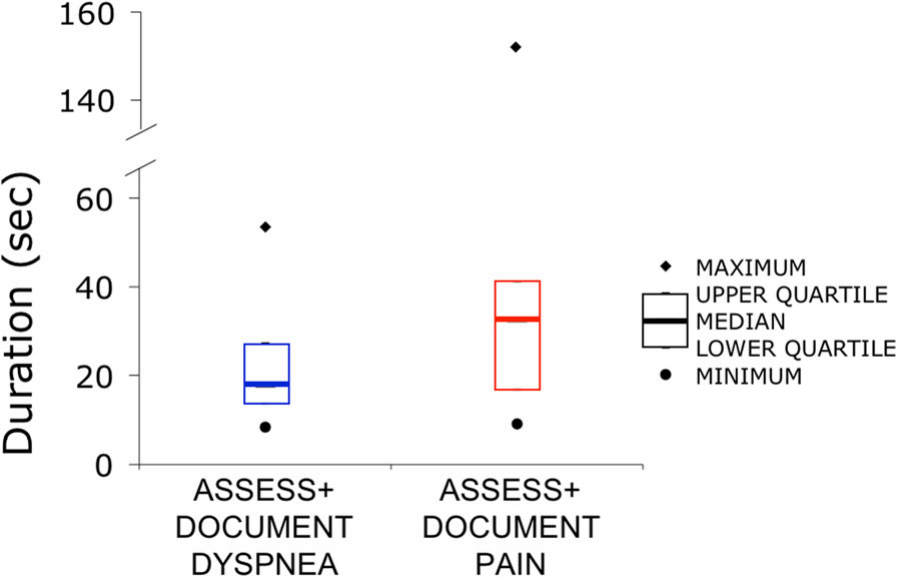
Time motion data – Heavy line denotes median time; box encompasses upper and lower quartiles; open diamond denotes maximum and open circle denotes minimum

The time motion study revealed that it took less than a minute to assess and document dyspnea, comparable to pain (Fig. 5). Nurses in the focus sessions reported that the only time there was burden on workflow was when patients experience clinical deterioration; but that this was not a reflection of the addition of the dyspnea scale but of a change in patients’ condition.

### Processes nurses use for assessment of dyspnea

Typically, nurses first asked a patient a Yes/No question, e.g., “are you short of breath?” If the answer was “no”, most nurses skipped asking for a rating and recorded “0” on the flowsheet (Additional file 17: Figure S14 a & b). If ‘yes’, the nurse often proceeded to obtain a patient rating. Nurses commonly use these phrases to explain to the patient what they were supposed to rate: most often “difficulty/trouble breathing” or “short of breath” and less commonly “breathing discomfort” or “can’t catch your breath” (Additional file 18: Figure S15).

**Fig. 6:**
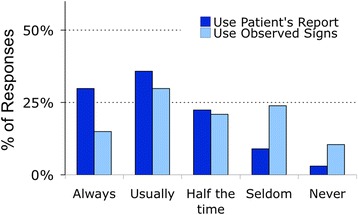
Nurses’ use of patient report vs observed signs for scaling dyspnea

Nurses reported using both the patient’s self-report and the nurse’s observation of the patient when documenting dyspnea (Fig. 6). The large overlap in the data shows that nurses often use both the patient’s report and their own observations to arrive at a number to document. (Of the 44 nurses who ‘always’ or ‘usually’ used patient report, 20 also reported ‘always’ or ‘usually’ using observed signs; conversely, of the 30 nurses who ‘always’ or ‘usually’ used physical signs, 20 reported ‘always’ or ‘usually’ using patient report.) (Additional file 19: Figures S16 & Additional file 20: Figure S17).

**Fig. 7:**
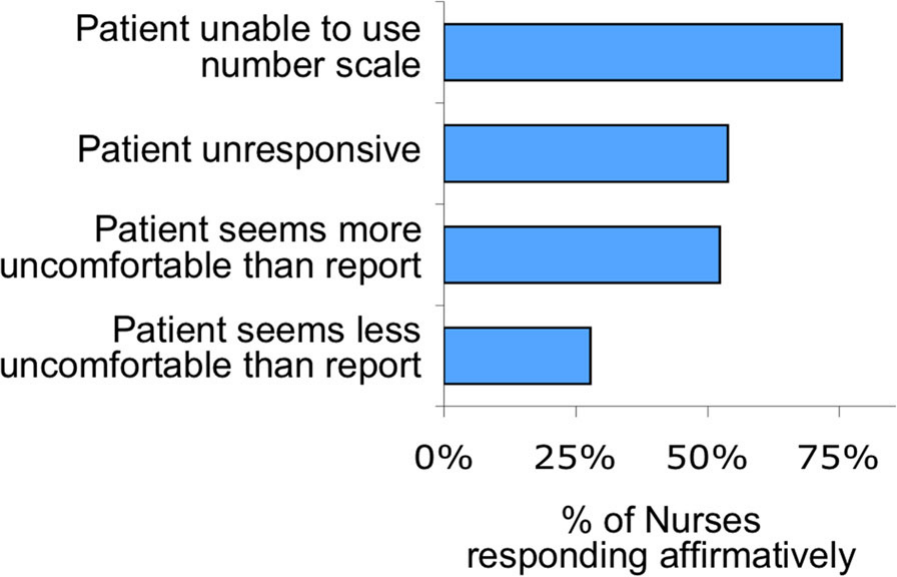
Reasons that nurses used signs rather than patient report

The most common reasons cited for using observed signs were sedation or the patient’s inability to use a number scale. Nurses sometimes used physical signs if their observations differed from the patient’s reported rating; when doing so they tended to err on the side of patient comfort: 52% of nurses reported that they used signs when the patient appeared more uncomfortable than patient report indicated, while only 28% used signs when the patient appeared less uncomfortable than patient report indicated (Fig. 7).

Nurses used several signs to infer dyspnea: tachypnea, difficulty speaking, accessory muscle use, nasal flaring, and restless movements were behaviors used frequently to assess for distress; heart rate and fearful facial expression were signs used less often (Additional file 21: Figure S18). Following their physical and verbal assessments, the nurses reported asking patients to rate their breathing using the 0-10 scale. Many equated the dyspnea scale to the pain scale and felt it was logical to assess for both dyspnea and pain together, as one nurse stated:

*“I usually do it after the pain score, if you ask about pain, then give them the scale (0 to 10), then repeat the same thing with the dyspnea rating.”*

### Patients’ ability to rate breathing discomfort using the 0–10 scale

Many nurses feel that rating dyspnea using the 0–10 scale is easy for alert and oriented patients; in some cases, they reported in the focus groups that “*rating of dyspnea was easier than rating pain*” and “*it’s easier for them to understand than the pain scale.*” One nurse explained that she could rely on patients’ self-rating of their dyspnea:

*“I feel people usually get* [i.e., understand] *shortness of breath. I feel like people give me a more accurate rating of their shortness of breath than of pain.”*

Nurses reported using the suggested words on the 0-10 scale to guide patients in their self-rating (none, mild, moderate, severe or unbearable) (Fig. 1).

*“I ask if they have trouble breathing; if yes, then I ask if it is mild/moderate, and then I ask “would you say it is a 5?” Then the patient says yes.”*

**Fig. 8:**
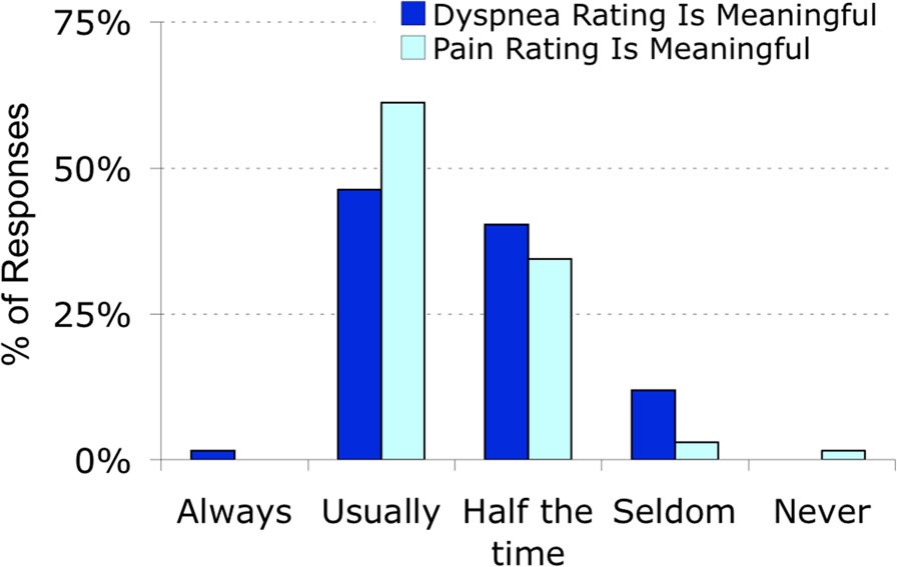
Nurses’ opinion of patients’ ability to provide meaningful rating of pain or dyspnea

When comparing patients’ ability to rate pain vs dyspnea, nurses had only slightly less confidence in patient’s ability to provide a meaningful number rating for dyspnea than for pain (Fig. 8, Additional file 22: Figure S19 & Additional file 23: Figure S20). In some instances, however, the nurses reported that they were unsure that patients understood the dyspnea scale even after explaining and clarifying. Difficulty in dyspnea assessment arose in patients who had cognitive impairment or who were not fluent in English. In these cases, the nurses reported using signs to assess patients’ breathing and assigning a number or indicating dyspnea with a “yes/no” or other sign.

**Fig. 9:**
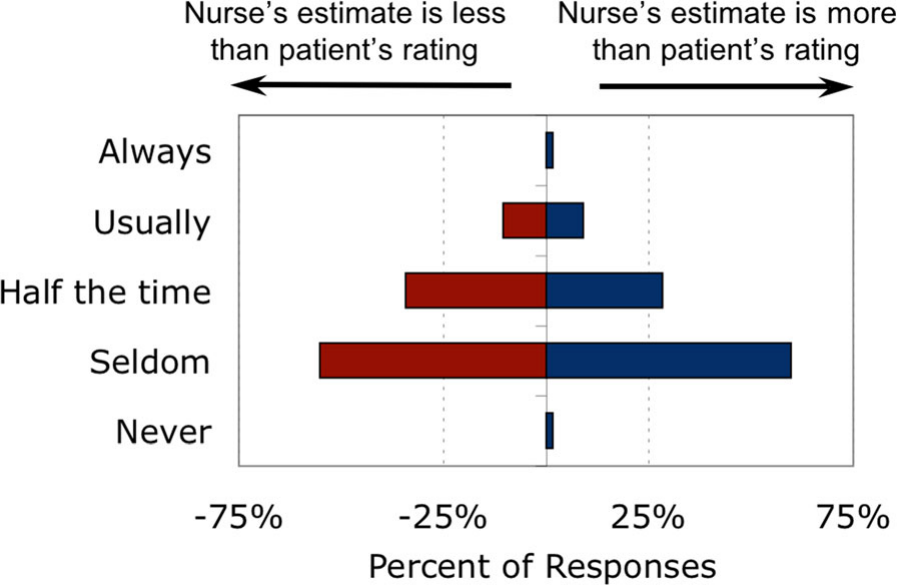
Nurse underestimates and overestimates when using signs to infer dyspnea

Nurses did report there were times they doubted patients’ self-reported dyspnea score, stating patients either appeared more or less uncomfortable than the rating (Fig. 9, Additional file 24: Figure S21 & Additional file 25: Figure S22). One nurse in the on-line survey who reported patients “usually” seem to have more respiratory distress than indicated by his/her rating added: *“on the written flowsheet sometimes the dyspnea score will be zero however the patient is exhibiting signs of distress”*.

### Nurses’ suggestions for dyspnea assessment

#### Assess patients for exertional dyspnea

Some nurses pointed out in the focus groups that assessing dyspnea while the patient is at rest did not give a complete picture of the patient’s condition. They suggested that assessing dyspnea with exertion would provide a better indication of the patient’s condition (i.e., increased shortness of breath with ambulation), as exemplified by these comments:

*“People tend to not have shortness of breath when they are lying down, but after physical therapy, they have shortness of breath.”*

*“They might say a ‘3’* [on initial assessment] *but when they get up to walk it’s an ‘8’.”*

#### Request for a pictorial scale

Although most nurses found the scale adequate, some suggested that a picture scale, similar to those developed for pain assessment Palos et al. [34], might help patients use the scale. One nurse remarked in the focus group session, *“They* [patients] *can’t relate it* [the number scale] *to how they are breathing so I have to coach them* [on how to use the scale]*. I wish there were pictures like the pain scale or more adjectives.”*

#### Request for systematic observation scale

Many nurses in both the focus group sessions and survey reported using observed physical signs of respiratory distress to help provide a number for dyspnea on the patient care flowsheet, stated by these nurses:

*“The scoring system should ask whether accessory muscles are involved, whether the respiratory rate is greater than normal, etc. There should be physical characteristic assessment questions involved in deriving…the ultimate number.”*

*“Maybe for a population that can’t speak, we* [could] *have some other kind of scale, something like the pain scale where you can see clinical* [signs]*, like the FLACC* (Faces, Legs, Activity, Cry, Consolability) *scale, or looking at a patient, we can give them a number.”* (FLACC scale [8])

## Discussion

Our data show that assessment and documentation of dyspnea took less than a minute of a nurse’s time and the overwhelming majority of nurses reported that it is easy to do. Many considered it an important improvement for patient-centered care.

### Nursing acceptance

#### Perceived importance

Nurses understood the importance of routinely measuring dyspnea and were very supportive of using a uniform tool to assess and document upon admission and every shift. Nurses further stated that implementation of routine dyspnea measurement increased their awareness of dyspnea. Our survey showed that nurses endorsed the idea that routine assessment can improve management and relieve suffering in patients. In fact, nurse adherence to the every-shift assessment of dyspnea was 86%, equivalent to reported adherence rates for pain assessment [9–14].

#### Effect on workflow

Quantitative measurement of dyspnea is not performed routinely at most hospitals. A survey of hospitalists, regarding only patients admitted for acute cardiopulmonary disease, suggested that the addition of dyspnea assessment would “have a significant effect on existing nursing and physician workflows” [4]. However, our data show the majority of clinical nurses readily adopted routine dyspnea measurement on *all* patients, finding it easy to incorporate the new documentation into their workflow. Routine assessment and documentation did not hinder workflow.

Dyspnea assessment and documentation was easily accomplished by nurses, not just in high-risk patients, but in the general medical-surgical population. Nurses often stated that measurement of dyspnea has always been a part of patient assessment, but the use of a standard tool to assess actually improved workflow and standardized documentation was more easily followed by colleagues.

### Why universal assessment?

Nurses reported that a standardized, documented dyspnea rating improved tracking of patient condition. The ability to trend a change in dyspnea over time may provide actionable information on patient clinical decline; lead to early interventions, ascertain whether interventions were helpful, and decrease symptom burden. Dyspnea assessment is of obvious importance in cardiopulmonary diseases and advanced cancer, but all hospitalized patients are at increased risk of cardiopulmonary issues such as pulmonary embolus and hospital-acquired pneumonia. For instance, pulmonary embolism carries a high fatality rate: 4.2% in hospital and 13.8% at 90 days after hospital discharge [15]. These events often occur in patients admitted for non-cardiopulmonary disorders including pregnancy, cancer, surgery, and trauma. There is preliminary evidence that a dyspnea score during hospitalization may be useful for predicting adverse outcomes in patients. Universal dyspnea assessment may reduce the risk that emerging or latent cardiopulmonary issues will be missed.

### The problem of symptom vs sign

#### The value of symptom reports

Expert statements emphasize that patient self-report of symptoms such as dyspnea and pain is the single most reliable measure of symptom intensity [3, 16]. The majority of nurses we surveyed agreed that patients usually give meaningful ratings of both dyspnea and pain. Although pain ratings are much more familiar, nurses have only slightly less faith in dyspnea ratings than in pain ratings (Fig. 8).

Although patient self-reports can provide the best information, they are not a perfect gold standard. Our studies using controlled laboratory stimuli have shown that about 85% of healthy individuals are able to reliably scale respiratory discomfort [17, 18]. There are, however, several reasons that an individual patient’s report of symptoms may not be an accurate reflection of that patient’s primary sensation [19]. We also know that dyspnea, like pain, is a multidimensional experience – when patients are asked to rate pain or dyspnea on a one-dimensional scale, they may focus on (hence rate) different aspects of the experience [20]. There are two recently developed instruments that incorporate multiple dimensions [21, 22]. Such instruments may be useful for follow-up in problematic cases, but single-dimension scales are better suited for the task of routine administration by busy nurses in an acute care setting.

Despite “subjective” distortions, patients’ reports of dyspnea are the best measure of what the patient feels, and they have proven very effective in predicting objective outcomes, particularly predicting survival in patients with chronic obstructive pulmonary disease (COPD), cancer, and cardiac disease [23–27].

#### Use of signs as a proxy for symptoms

“When the patient is able to report pain, the patient’s behavior or vital signs should never be used in lieu of self-report’ [16]; this principle also applies to dyspnea [3]. Although this concept was emphasized in training, many nurses reported using their observations of physical signs to modify or replace the patient’s report of dyspnea (Fig. 6). Unfortunately, the structure of our questionnaire does not allow us to understand fully how nurses incorporated their observation of signs with the patient’s symptom report.

Although nurses usually used signs appropriately (in sedated patients or patients unable to understand the concept of numerical rating), they sometimes used signs when they were skeptical about the reliability of patients’ ratings. Studies in which patients’ ratings of current dyspnea have been compared with concurrent physicians’ and nurses’ inference of dyspnea from observed signs have shown poor concordance as indicated by Cohen’s kappa for inter-rater agreement < 0.2 and/or coefficient of determination, r^2^, <0.2 [28, 29]. These studies show that both nurses and physicians tend to greatly underestimate dyspnea most of the time. Despite poor agreement on the individual level, health care professionals’ estimates are better than nothing at all in the case of the patient who is unable to communicate.

Some nurses asked for a more structured method to infer dyspnea from signs, and one such instrument has recently been developed, the 8-item Respiratory Distress Observation Scale (RDOS) [30]. However, in the only study comparing RDOS scores with unstructured observation, the RDOS did not perform as well as unstructured observation by bedside nurses [29]. Further refinement of the RDOS may improve concordance with patient ratings [31], and the instrument may help to educate inexperienced nurses and physicians.

Although published studies show a tendency for nurses observing the patient to provide a lower rating for dyspnea than the patient, nurses in our study did not feel that patients were rating their dyspnea “too high” (Fig. 9). The nearly neutral bias in our survey may result from nurses learning to “calibrate” their observations by repeatedly asking patients for dyspnea ratings.

### Improving dyspnea assessment

#### Provide space for reporting observation of signs

Our focus group discussions with nurses suggested that when they used observed signs to inform the documented dyspnea rating, they were not simply ignoring the concept that the patient’s discomfort is what the patient says it is. Rather, the nurses were attempting to report suffering or distress that they inferred from observation when they felt the patient was unable to provide a report, or unwilling to admit suffering. We suggest that a major improvement to the dyspnea assessment process would be to add an optional second scale for nurses to record their observation of respiratory distress based on signs. This would 1) reduce the tendency for nurses to modify the patient’s report, 2) provide additional clinically useful information, and 3) simplify the nurse’s task because s/he would not be forced to decide whether to report symptom or sign.

#### Nurses should not skip scale after yes-no question

More than half the nurses skipped asking for a numerical rating if the patient denied breathing discomfort in a preliminary yes-no question. This procedure saves a few seconds, but is less than optimal. Binary responses to a query about the presence of a discomfort require the patient to make a decision about how much discomfort merits a ‘yes’ response; an answer of “no” is often given for values above zero on a rating scale. The level of sensation required to elicit a “yes” response is known as the ‘decision criterion’, a phenomenon well known in psychophysics [32]. Our experience is that patients will often follow a ‘no’ response with a non-zero rating. We suggest that if the patient answers ‘no’, that the nurse continues to the scale with a transition such as “Even though you said ‘no’, it would be helpful to us if you could indicate your pain on this scale so that we can track it easily from one time to the next.”

#### Document exertional dyspnea

During our focus groups, nurses reported that the every-shift dyspnea assessment performed while the patient is at rest does not fully capture patients’ dyspnea during activities, such as rising to use the lavatory or working with physical therapy. Patients experiencing dyspnea limit their activities to minimize symptom burden. Nurses felt that assessment could be improved by capturing patients’ breathing discomfort with various activities throughout the day. The numeric scale is easy to administer both at rest and with activity. We suggest that dyspnea assessment could be improved by measuring breathing discomfort during periods of exertion, and recording the nature of activity that produced the rating – similar to our IPA.

### Symptom management

Nurses at our institution feel severe dyspnea should be treated and that an algorithm with possible treatment modalities would be helpful (Additional file 26: Figure S23). An increase in dyspnea can be a warning of worsening condition, so the first response should be to discover the underlying problem, and if possible address it. This is an opportunity for integrated care, and the surveyed nurses reported that physicians took appropriate actions in the large majority of cases (Additional file 27: Figure S24a-f). When underlying causes have been addressed and dyspnea persists, there are effective strategies to alleviate dyspnea, including repositioning, use of a facial fan, providing reassurance, and use of opioid therapy [33]*.*

### Study limitations

The study was conducted at a single academic tertiary care hospital, and may not generalize to all care settings. The questions for the survey were investigator developed and some questions could have been worded differently for improved clarity. For example, the question “do patients give a meaningful number rating for dyspnea?” did not separately account for patients with cognitive impairment. We were unable to capture all information during the focus groups sessions (e.g., nods of agreement). For this reason, the quantitative data in this report rely entirely on the randomized anonymous survey.

## Conclusion

Patient self-report has long been the standard for assessment and management of pain. Lack of routine dyspnea assessment and documentation is a barrier to improving management of dyspneic patients. Our data show that nurses endorsed routine dyspnea documentation, and did not find it burdensome.

Dyspnea is an under-recognized and distressing symptom. Prior to our standardization of assessment, dyspnea was documented inconsistently in our hospital. The numeric/word scale, though unidimensional, is fast, patient-focused, and easy to administer at rest or with activity. Dyspnea, like pain, is what the patient says it is and must be treated as such. Nurses caring for patients must promptly assess dyspnea and intervene to minimize this frightening sensation. Only then will we have the ability to relieve suffering and improve patient centered care through symptom management.

## Additional files

Additional file 1: Table S1. On which unit do you currently work? Demographic information. (TIF 58 kb)
Additional file 2: Table S2. What ethnicity do you identify with? Demographic information. (TIF 28 kb)
Additional file 3: Table S3. What race do you identify with? Demographic information. (TIF 36 kb)
Additional file 4: Figure S1. How many years have you practiced nursing? Demographic information. (TIF 79 kb)
Additional file 5: Figure S2. Age distribution of nurses surveyed Demographic information. (TIF 101 kb)
Additional file 6: Figure S3. Have you attended a BIDMC-sponsored nursing focus group on dyspnea within the past 2 years? Assessment of prior training. (TIF 67 kb)
Additional file 7: Figure S4. Have you received educational training on dyspnea and the new dyspnea assessment at BIDMC? Assessment of prior training. (TIF 54 kb)
Additional file 8: Figure S5. If yes, what type of education did you receive? Assessment of prior training. (TIF 68 kb)
Additional file 9: Figure S6. How important is it to use a uniform tool to assess for dyspnea? Nurses’ perception of importance of dyspnea measurement. (TIF 70 kb)
Additional file 10: Figure S7. How important is it to track dyspnea every shift? Nurses’ perception of importance of dyspnea measurement. (TIF 63 kb)
Additional file 11: Figure S8. How important is it to assess dyspnea on admission? Nurses’ perception of importance of dyspnea measurement. (TIF 71 kb)
Additional file 12: Figure S9. How important is it to document dyspnea on admission? Nurses’ perception of importance of dyspnea measurement. (TIF 69 kb)
Additional file 13: Figure S10. How important is the addition of routine dyspnea assessment on improving patient-centered care? Nurses’ perception of importance of dyspnea measurement. (TIF 77 kb)
Additional file 14: Figure S11. How important is the addition of routine dyspnea assessment in predicting adverse patient outcomes? Assessment of nurses’ perception of importance of dyspnea assessment. (TIF 74 kb)
Additional file 15: Figure S12. How easy or difficult is it to administer the dyspnea assessment? Effect of routine dyspnea assessment on nursing workflow. (TIF 66 kb)
Additional file 16: Figure S13. How has the implementation of Q-shift dyspnea assessment affected your workflow? Effect of routine dyspnea assessment on nursing workflow. (TIF 68 kb)
Additional file 17: Figure S14. a. Do you first ask a yes/no question when assessing dyspnea? Method nurses used to assess dyspnea. b. If the patient responds “no”, do you skip asking for a number and record “0” on the flowsheet? Method nurses used to assess dyspnea. (ZIP 101 kb)
Additional file 18: Figure S15. Which of the following words or phrases do you use to explain to the patient what they are supposed to rate (check all that apply)? Method nurses used to assess dyspnea. (TIF 108 kb)
Additional file 19: Figure S16. How often do you use *the patient’s report* to provide a number for dyspnea on the Patient Care Flowsheet? Method nurses used to assess dyspnea. (TIF 64 kb)
Additional file 20: Figure S17. How often do you use *observed physical signs of respiratory distress* to provide a number for dyspnea on the Patient Care Flowsheet? Method nurses used to assess dyspnea. (TIF 72 kb)
Additional file 21: Figure S18. When you use physical signs to assess respiratory distress, what signs do you use (check all that apply)? Method nurses used to assess dyspnea. (TIF 124 kb)
Additional file 22: Figure S19. In your opinion, do patients give a meaningful number rating for *pain*? Nurses’ perception of patient comprehension of questions. (TIF 63 kb)
Additional file 23: Figure S20. In your opinion, do patients give a meaningful number rating for *dyspnea*? Nurses’ perception of patient comprehension of questions. (TIF 67 kb)
Additional file 24: Figure S21. How often does the patient seem to have *less* respiratory distress than indicated by his/her rating? Nurses’ perception of reliability of patient rating. (TIF 73 kb)
Additional file 25: Figure S22. How often does the patient seem to have *more* respiratory distress than indicated by his/her rating? Nurses’ perception of reliability of patient rating. (TIF 67 kb)
Additional file 26: Figure S23. In your opinion, would it be useful to have an algorithm with specific options for the treatment of dyspnea (e.g., repositioning, facial fan, morphine)? Assessment of need for support with treatment options. (TIF 80 kb)
Additional file 27: Figure S24. a. When you report a patient’s dyspnea rating to the physician responsible for the patient, the physician requests vital signs and/or oxygen saturation. Nurses’ perception of physician response. b. When you report a patient’s dyspnea rating to the physician responsible for the patient, the physician orders laboratory or imaging studies. Nurses’ perception of physician response. c. When you report a patient’s dyspnea rating to the physician responsible for the patient, the physician orders an intervention to relieve dyspnea (pharmacologic or non-pharmacologic). Nurses’ perception of physician response. d. When you report a patient’s dyspnea rating to the physician responsible for the patient, the physician evaluates the patient. Nurses’ perception of physician response. e. When you report a patient’s dyspnea rating to the physician responsible for the patient, the physician requests nursing to reassess the patient later. Nurses’ perception of physician response. f. When you report a patient’s dyspnea rating to the physician responsible for the patient, the physician takes none of these actions. Nurses’ perception of physician response. (ZIP 467 kb)

## Abbreviations

BIDMC: Beth Israel Deaconess Medical Center
CNS: Clinical nurse specialist
COPD: Chronic obstructive pulmonary disease
FLACC: Faces, Legs, Activity, Cry, Consolability (non-verbal pain scale)
IPA: Initial Patient Assessment
iPAD Mini: Tablet brand used
IRB: Institutional Review Board
KMB: Kathy M. Baker (author)
NIH: National Institute of Health
RBB: Robert B. Banzett (co-author)
RDOS: Respiratory Distress Observation Scale
S: Supplement
SD-M: Susan DeSanto-Madeya (co-author)
