# Supplementary figures and images for: Routine dyspnea assessment and documentation: Nurses’ experience yields wide acceptance
Source: BMC Nurs. 2017 Jan 14;16:3. doi: 10.1186/s12912-016-0196-9 (PMC5237543; doi:10.1186/s12912-016-0196-9)

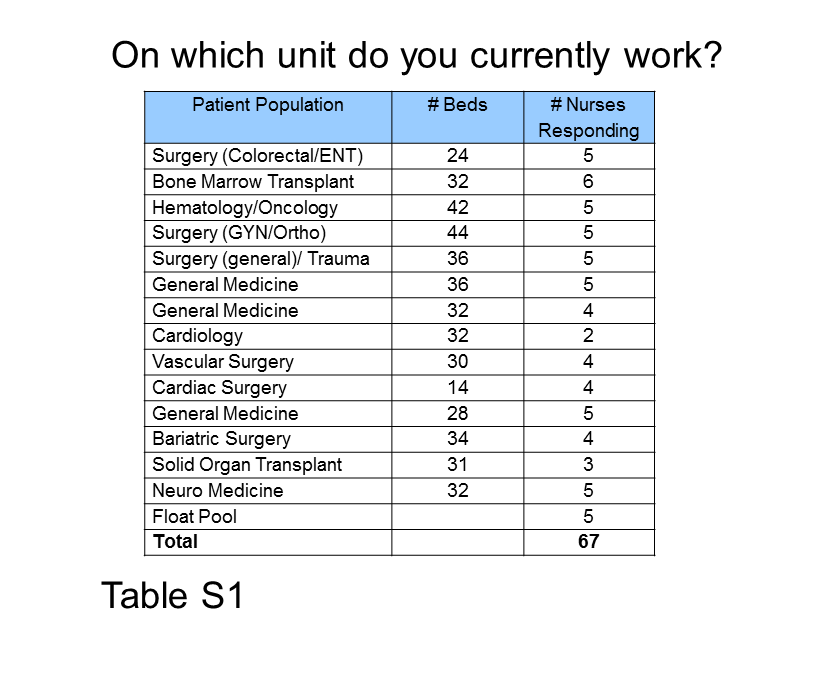

Supplement: Additional file 1: Table S1. — On which unit do you currently work? Demographic information. (TIF 58 kb) [file 12912_2016_196_MOESM1_ESM.tif]

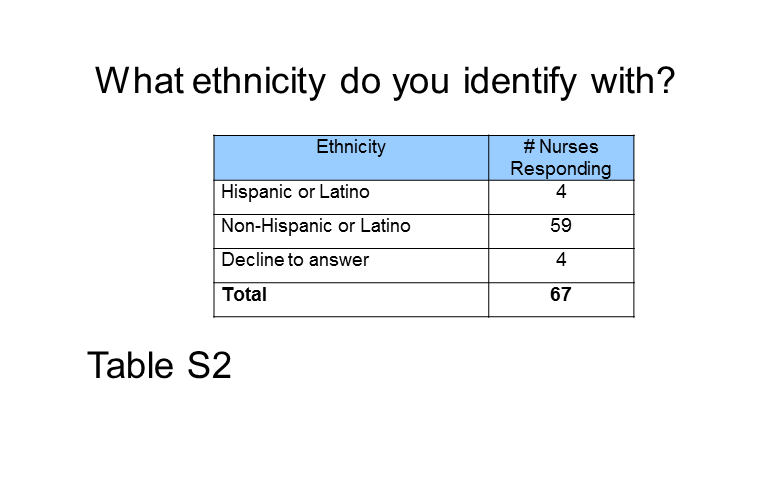

Supplement: Additional file 2: Table S2. — What ethnicity do you identify with? Demographic information. (TIF 28 kb) [file 12912_2016_196_MOESM2_ESM.tif]

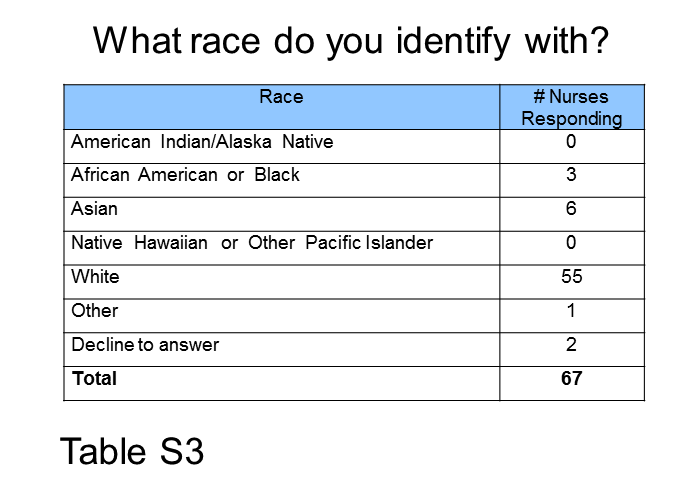

Supplement: Additional file 3: Table S3. — What race do you identify with? Demographic information. (TIF 36 kb) [file 12912_2016_196_MOESM3_ESM.tif]

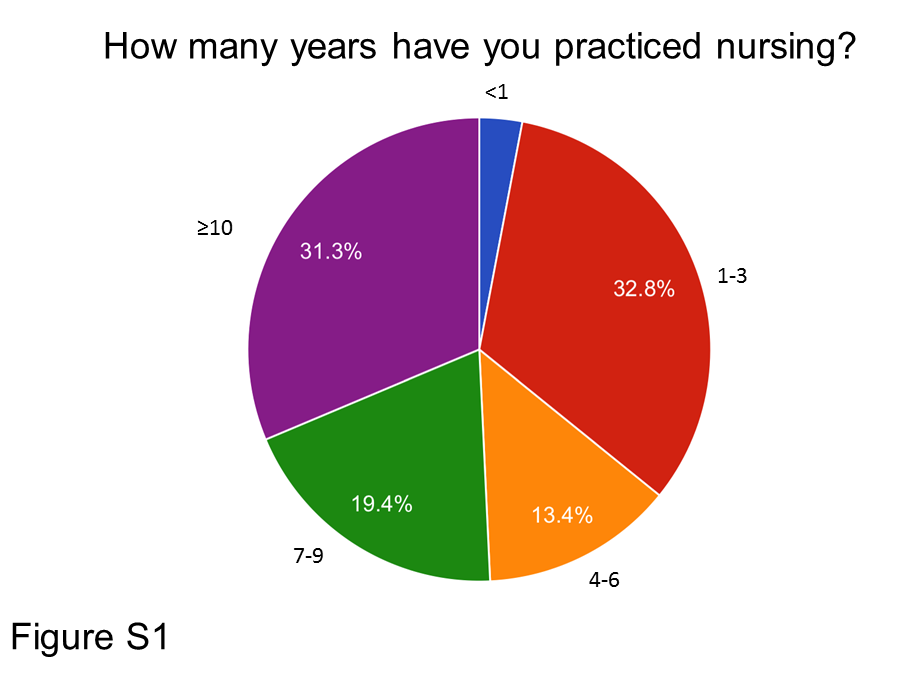

Supplement: Additional file 4: Figure S1. — How many years have you practiced nursing? Demographic information. (TIF 79 kb) [file 12912_2016_196_MOESM4_ESM.tif]

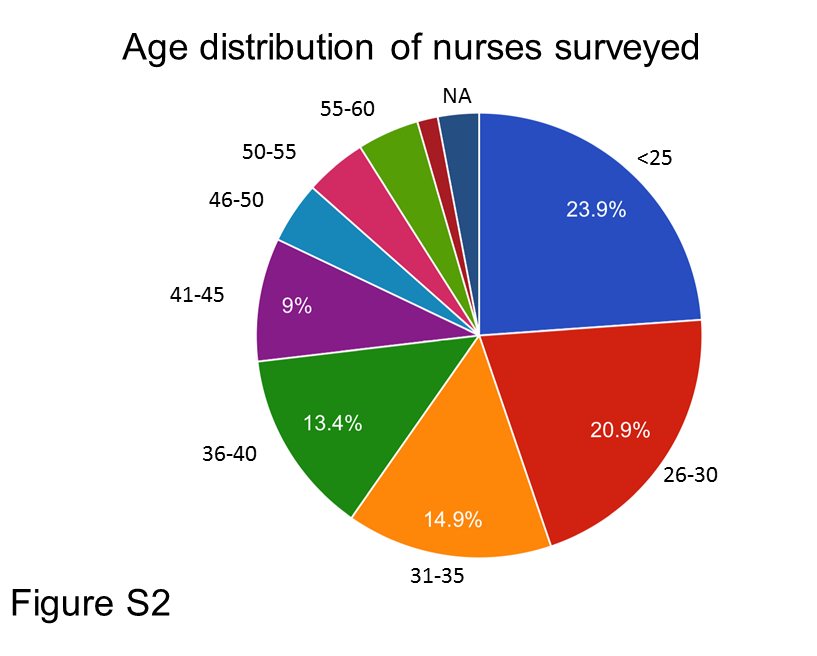

Supplement: Additional file 5: Figure S2. — Age distribution of nurses surveyed Demographic information. (TIF 101 kb) [file 12912_2016_196_MOESM5_ESM.tif]

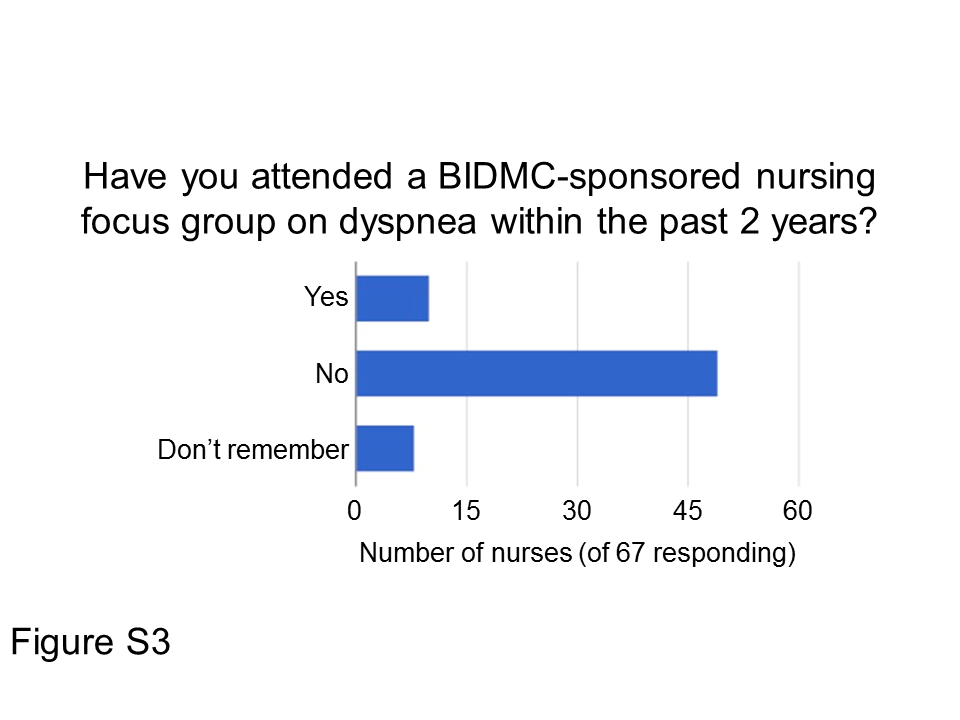

Supplement: Additional file 6: Figure S3. — Have you attended a BIDMC-sponsored nursing focus group on dyspnea within the past 2 years? Assessment of prior training. (TIF 67 kb) [file 12912_2016_196_MOESM6_ESM.tif]

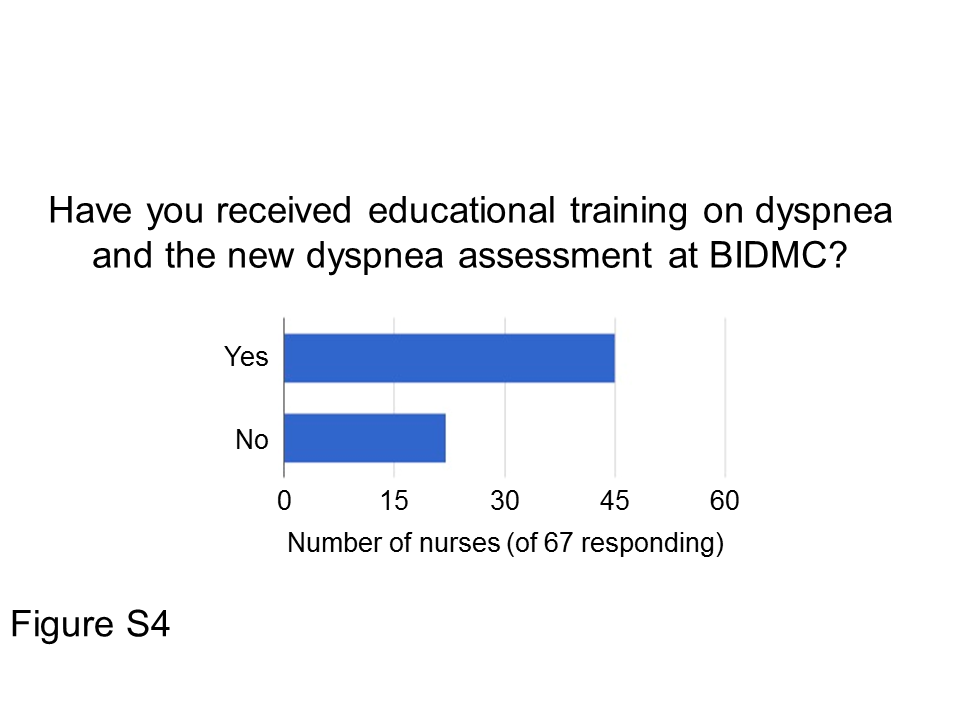

Supplement: Additional file 7: Figure S4. — Have you received educational training on dyspnea and the new dyspnea assessment at BIDMC? Assessment of prior training. (TIF 54 kb) [file 12912_2016_196_MOESM7_ESM.tif]

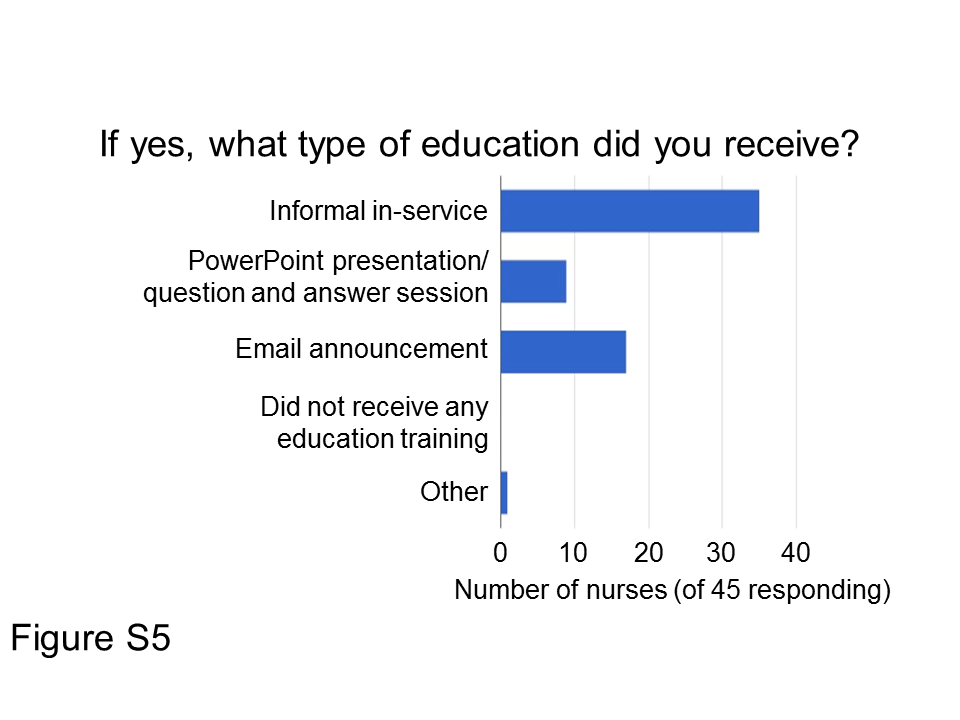

Supplement: Additional file 8: Figure S5. — If yes, what type of education did you receive? Assessment of prior training. (TIF 68 kb) [file 12912_2016_196_MOESM8_ESM.tif]

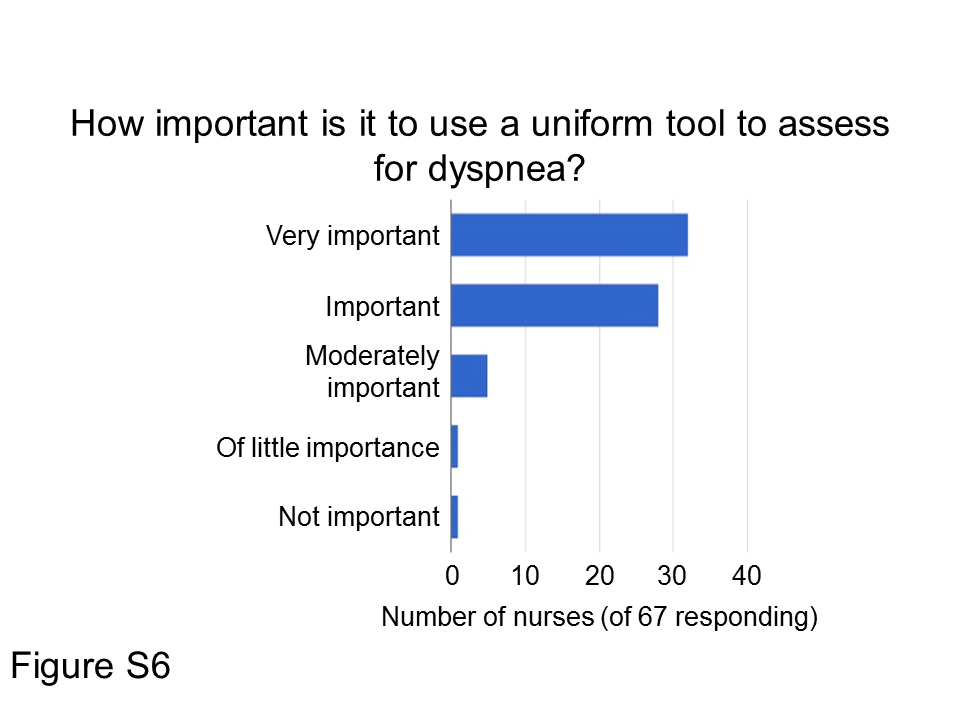

Supplement: Additional file 9: Figure S6. — How important is it to use a uniform tool to assess for dyspnea? Nurses’ perception of importance of dyspnea measurement. (TIF 70 kb) [file 12912_2016_196_MOESM9_ESM.tif]

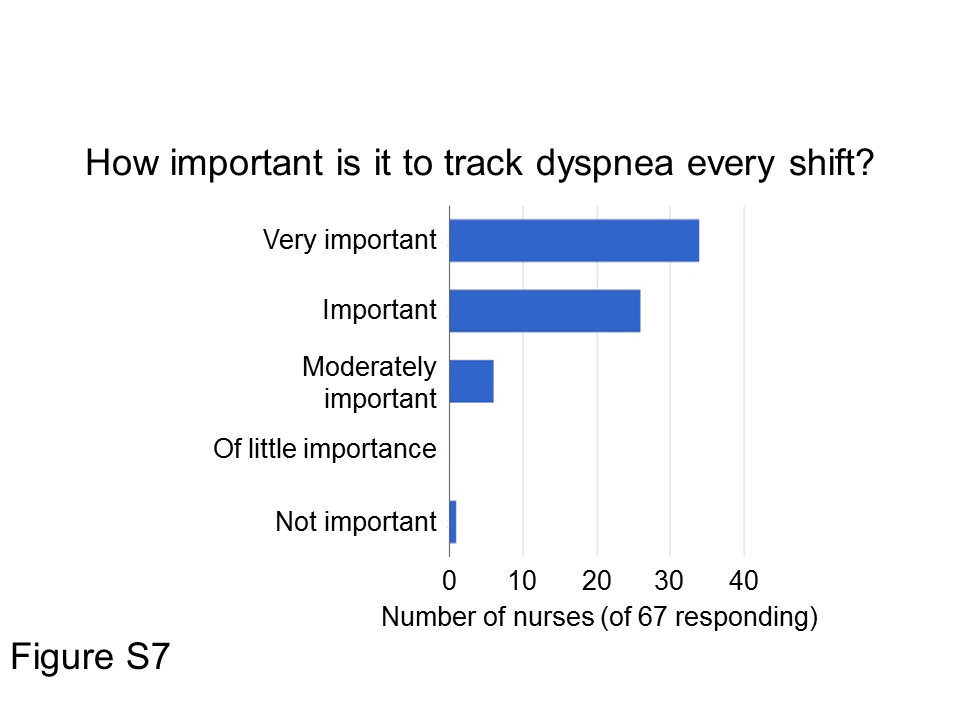

Supplement: Additional file 10: Figure S7. — How important is it to track dyspnea every shift? Nurses’ perception of importance of dyspnea measurement. (TIF 63 kb) [file 12912_2016_196_MOESM10_ESM.tif]

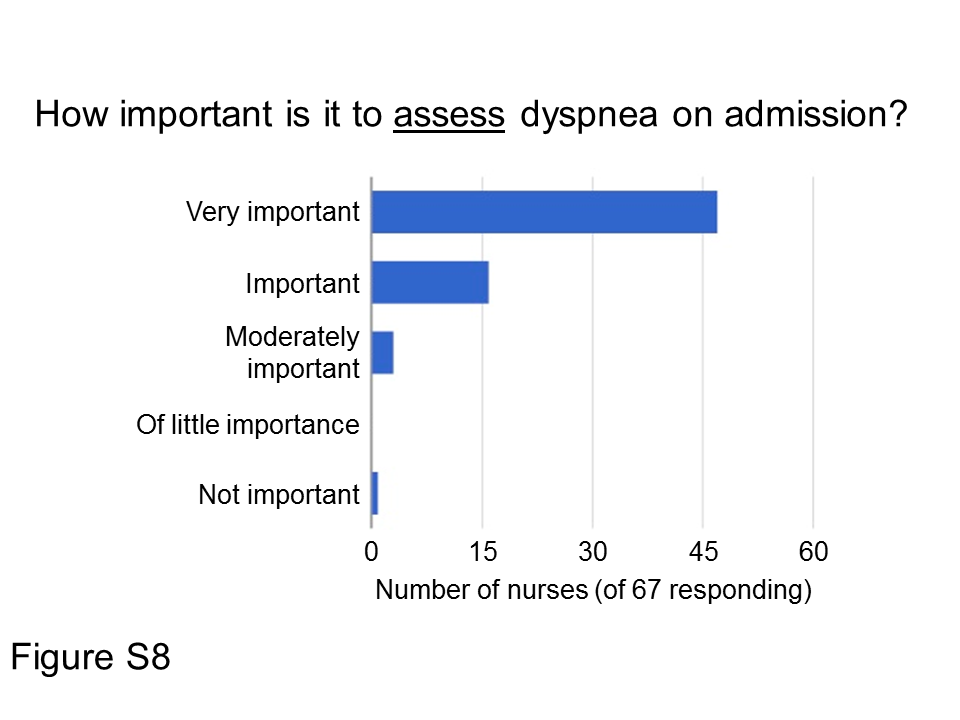

Supplement: Additional file 11: Figure S8. — How important is it to assess dyspnea on admission? Nurses’ perception of importance of dyspnea measurement. (TIF 71 kb) [file 12912_2016_196_MOESM11_ESM.tif]

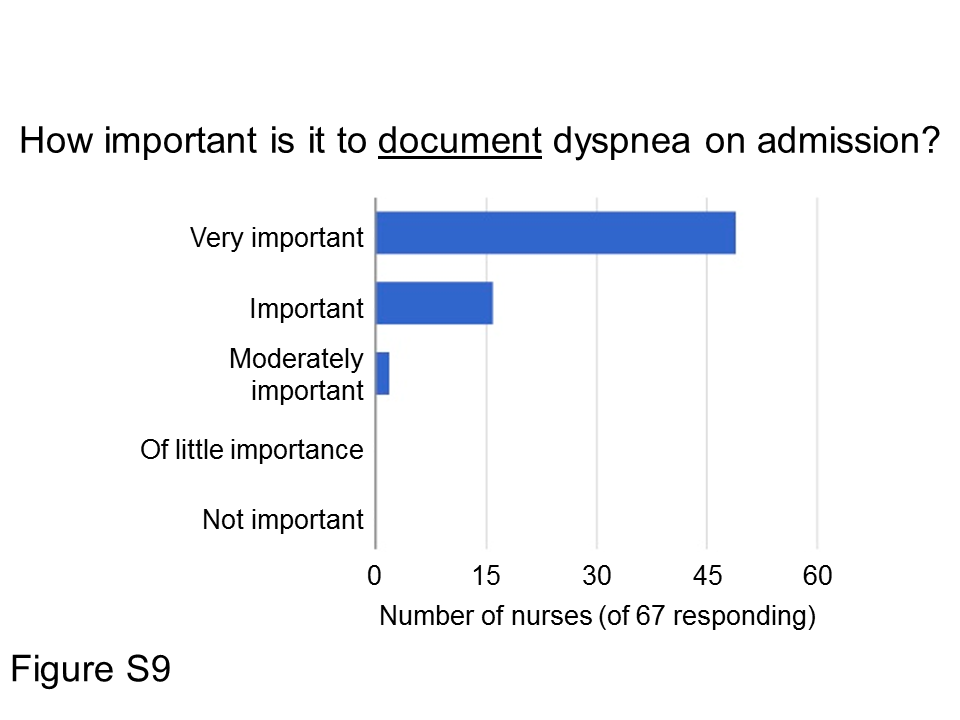

Supplement: Additional file 12: Figure S9. — How important is it to document dyspnea on admission? Nurses’ perception of importance of dyspnea measurement. (TIF 69 kb) [file 12912_2016_196_MOESM12_ESM.tif]

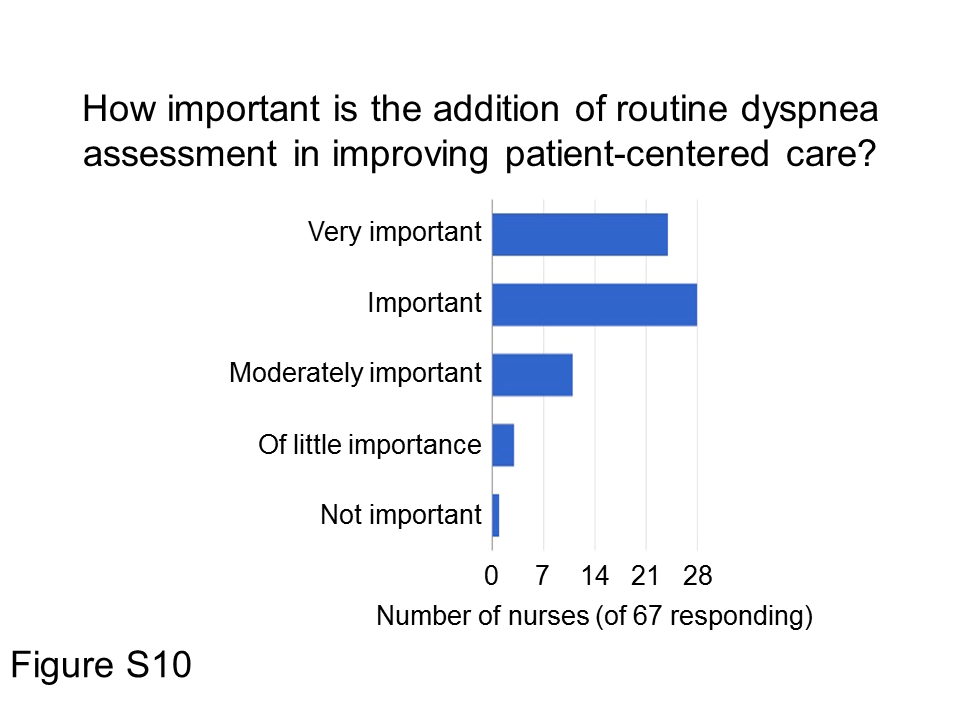

Supplement: Additional file 13: Figure S10. — How important is the addition of routine dyspnea assessment on improving patient-centered care? Nurses’ perception of importance of dyspnea measurement. (TIF 77 kb) [file 12912_2016_196_MOESM13_ESM.tif]

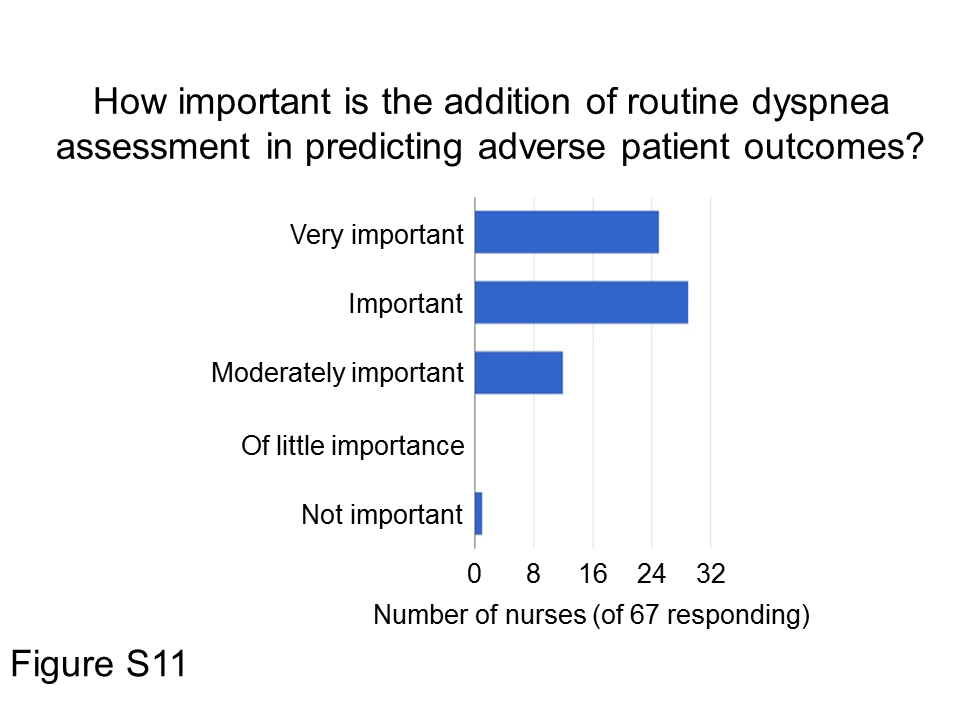

Supplement: Additional file 14: Figure S11. — How important is the addition of routine dyspnea assessment in predicting adverse patient outcomes? Assessment of nurses’ perception of importance of dyspnea assessment. (TIF 74 kb) [file 12912_2016_196_MOESM14_ESM.tif]

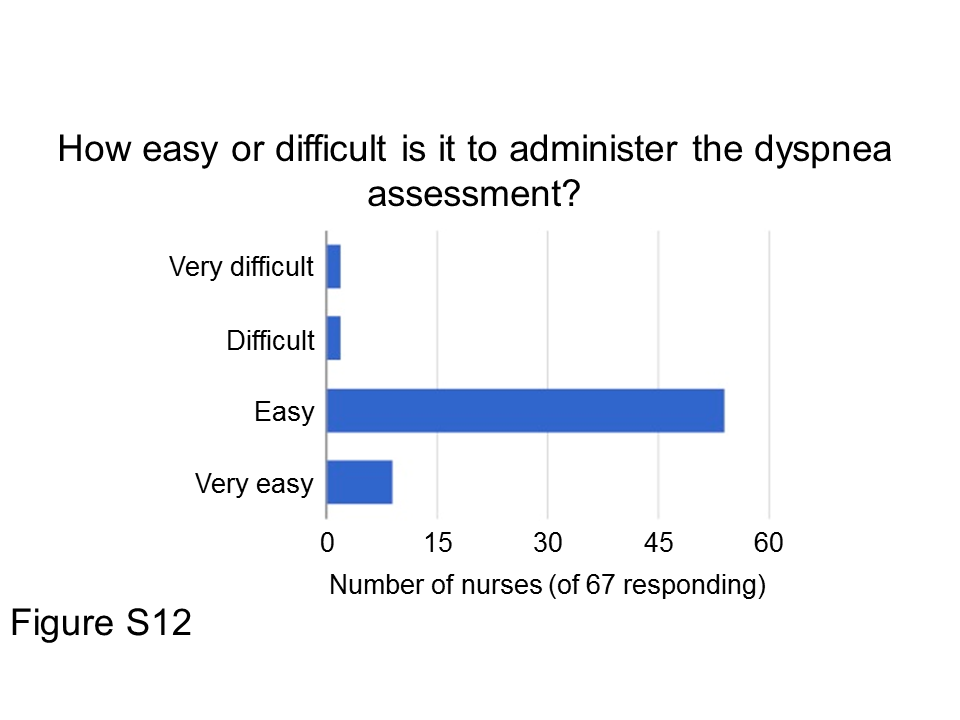

Supplement: Additional file 15: Figure S12. — How easy or difficult is it to administer the dyspnea assessment? Effect of routine dyspnea assessment on nursing workflow. (TIF 66 kb) [file 12912_2016_196_MOESM15_ESM.tif]

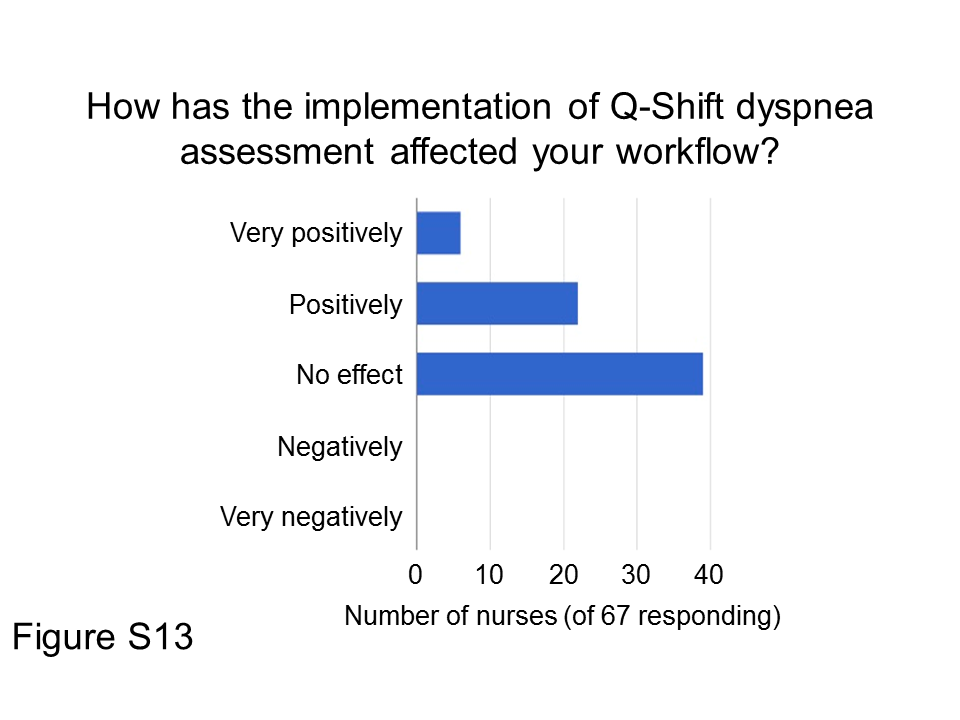

Supplement: Additional file 16: Figure S13. — How has the implementation of Q-shift dyspnea assessment affected your workflow? Effect of routine dyspnea assessment on nursing workflow. (TIF 68 kb) [file 12912_2016_196_MOESM16_ESM.tif]

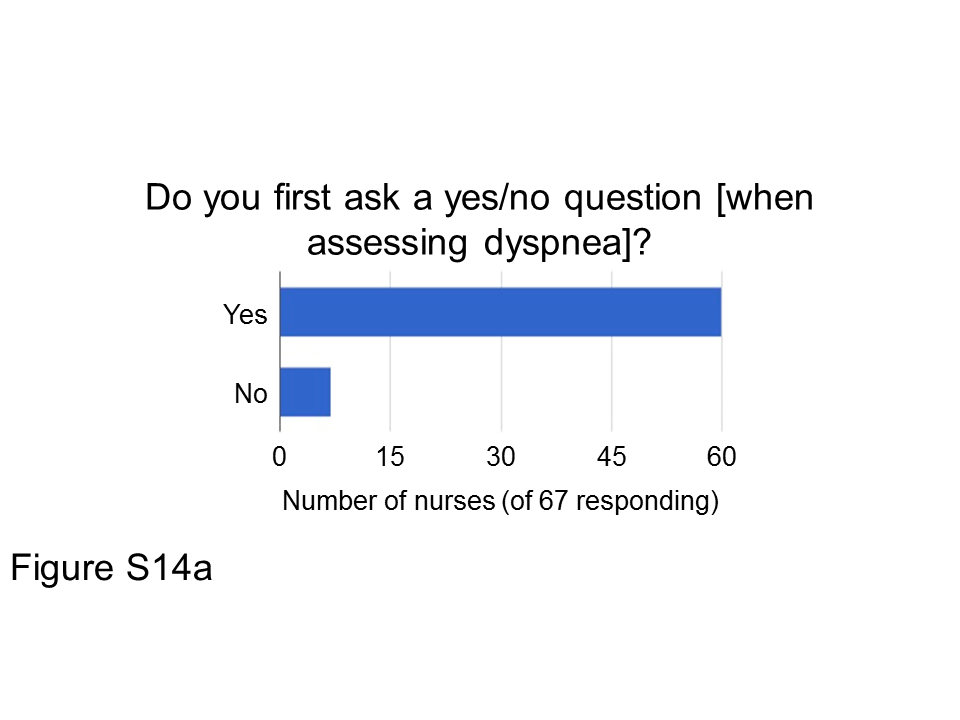

Supplement: Additional file 17: Figure S14. — a. Do you first ask a yes/no question when assessing dyspnea? Method nurses used to assess dyspnea. b. If the patient responds “no”, do you skip asking for a number and record “0” on the flowsheet? Method nurses used to assess dyspnea. (ZIP 101 kb) [file 12912_2016_196_MOESM17_ESM.zip › additional file 17/New S14aR2.tif]

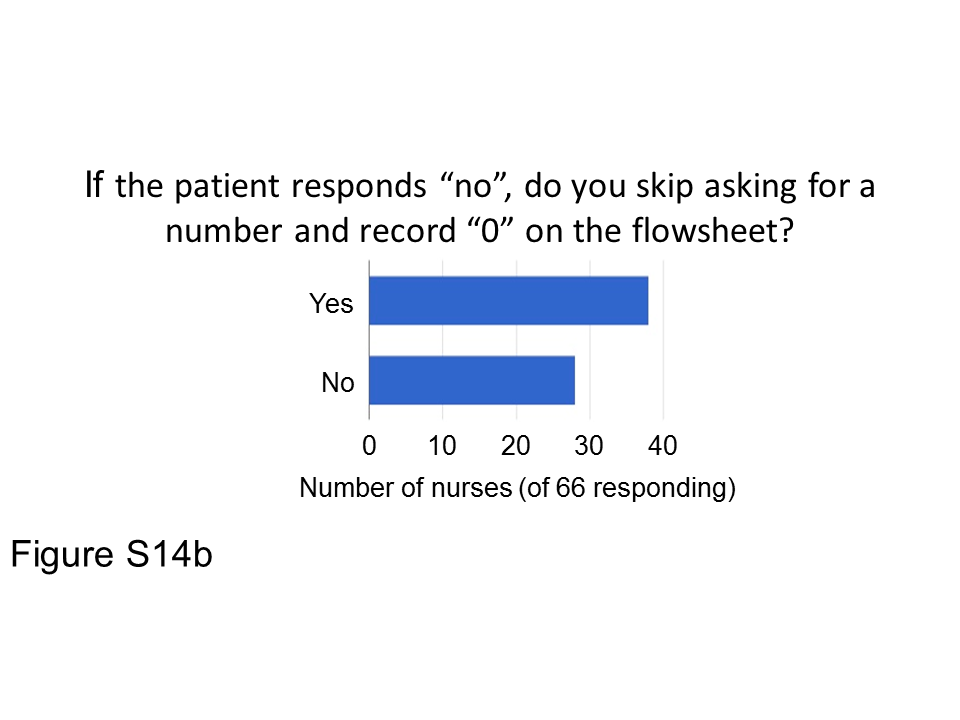

Supplement: Additional file 17: Figure S14. — a. Do you first ask a yes/no question when assessing dyspnea? Method nurses used to assess dyspnea. b. If the patient responds “no”, do you skip asking for a number and record “0” on the flowsheet? Method nurses used to assess dyspnea. (ZIP 101 kb) [file 12912_2016_196_MOESM17_ESM.zip › additional file 17/New S14bR2.tif]

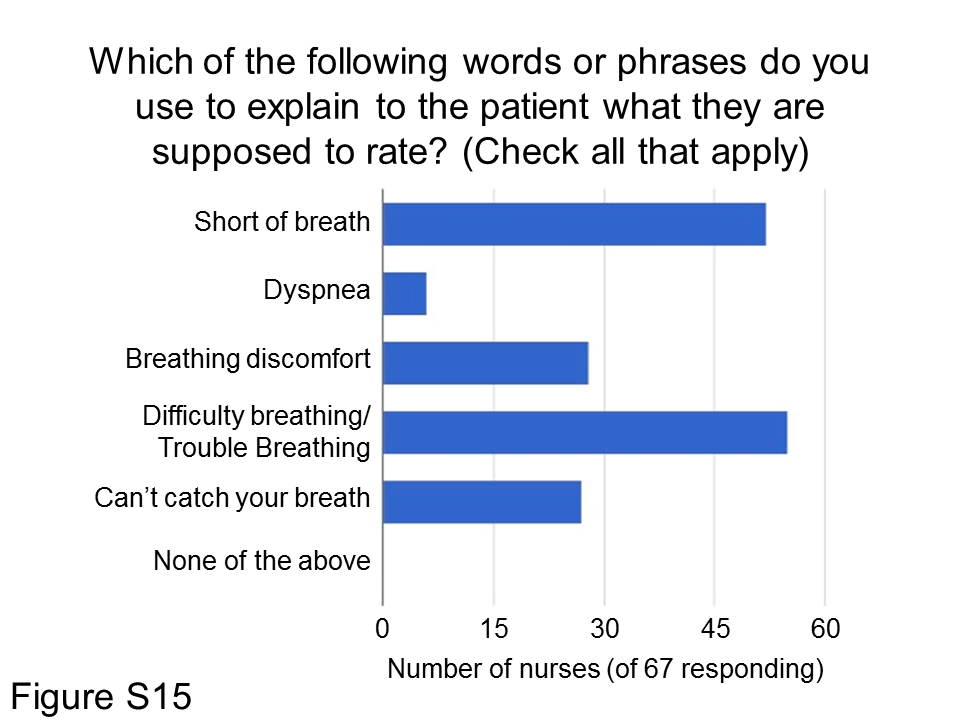

Supplement: Additional file 18: Figure S15. — Which of the following words or phrases do you use to explain to the patient what they are supposed to rate (check all that apply)? Method nurses used to assess dyspnea. (TIF 108 kb) [file 12912_2016_196_MOESM18_ESM.tif]

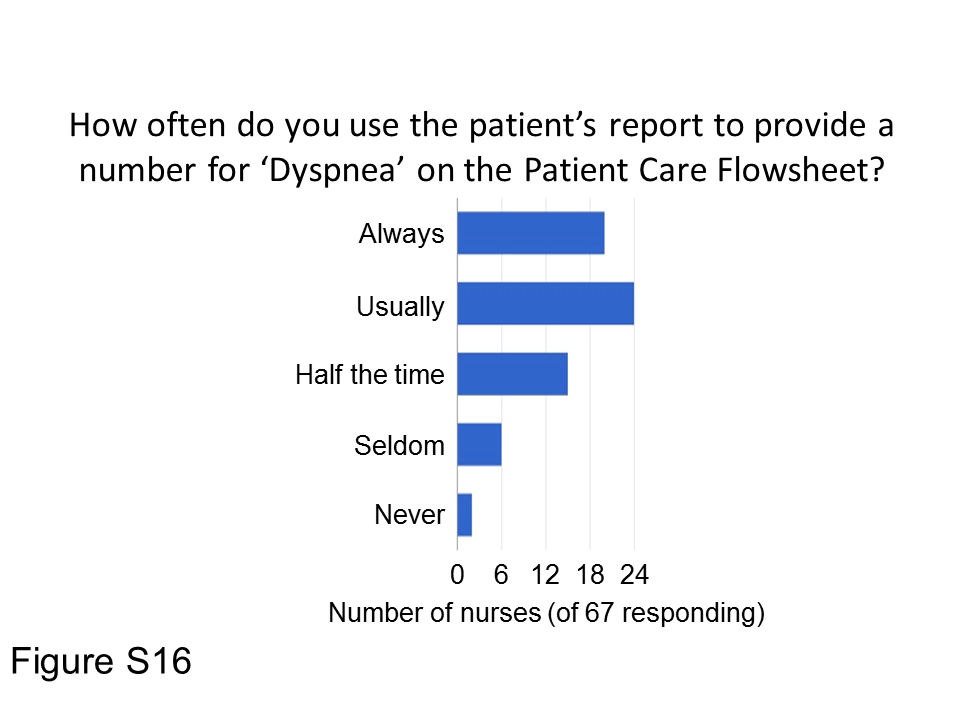

Supplement: Additional file 19: Figure S16. — How often do you use the patient’s report to provide a number for dyspnea on the Patient Care Flowsheet? Method nurses used to assess dyspnea. (TIF 64 kb) [file 12912_2016_196_MOESM19_ESM.tif]

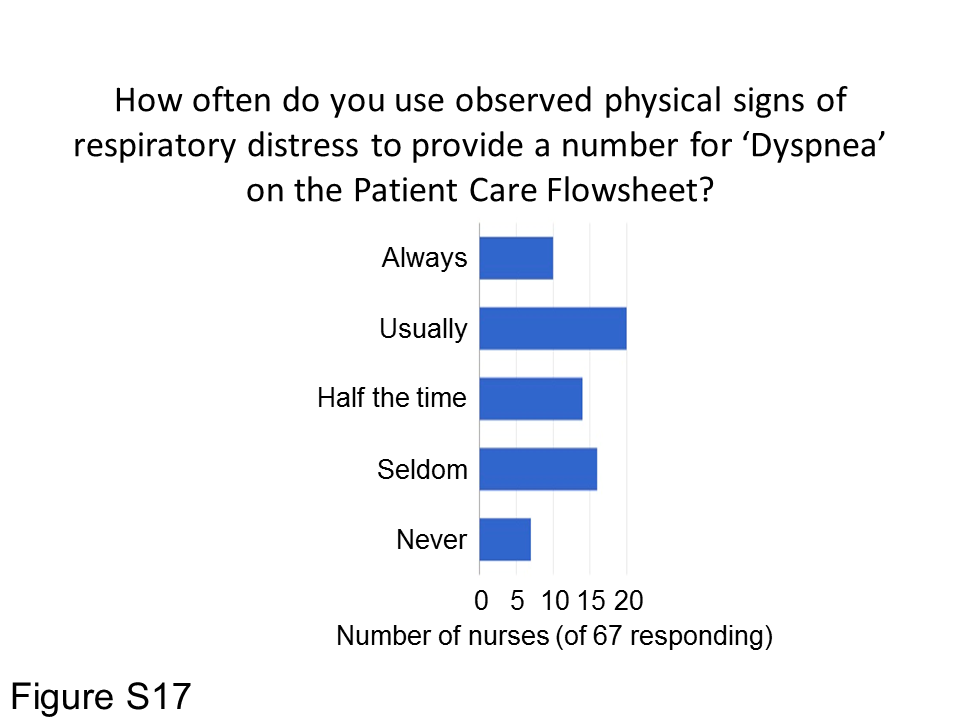

Supplement: Additional file 20: Figure S17. — How often do you use observed physical signs of respiratory distress to provide a number for dyspnea on the Patient Care Flowsheet? Method nurses used to assess dyspnea. (TIF 72 kb) [file 12912_2016_196_MOESM20_ESM.tif]

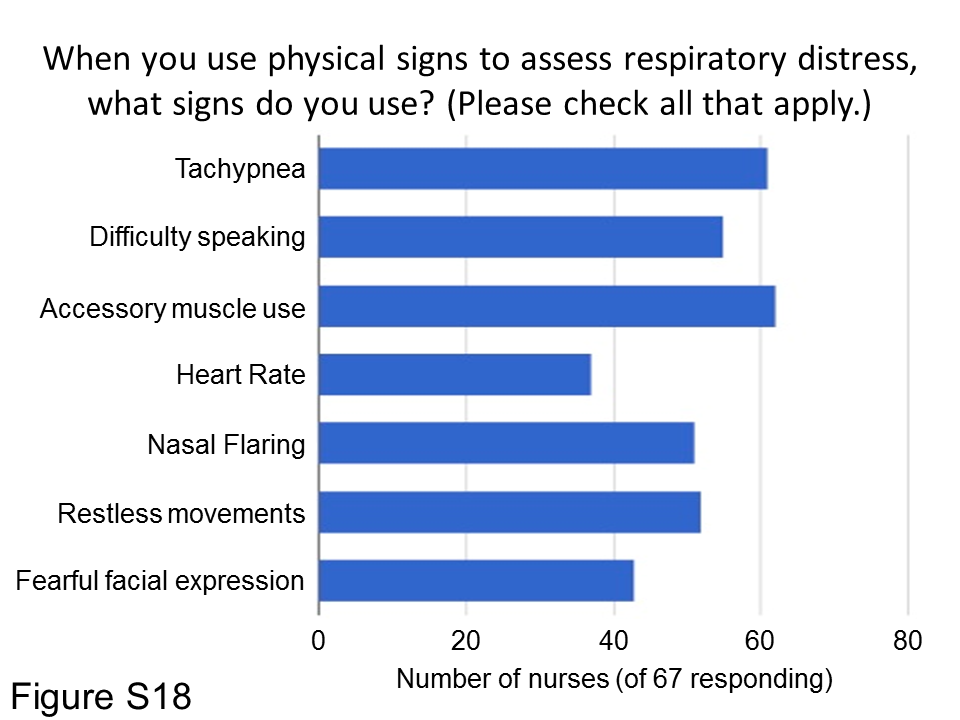

Supplement: Additional file 21: Figure S18. — When you use physical signs to assess respiratory distress, what signs do you use (check all that apply)? Method nurses used to assess dyspnea. (TIF 124 kb) [file 12912_2016_196_MOESM21_ESM.tif]

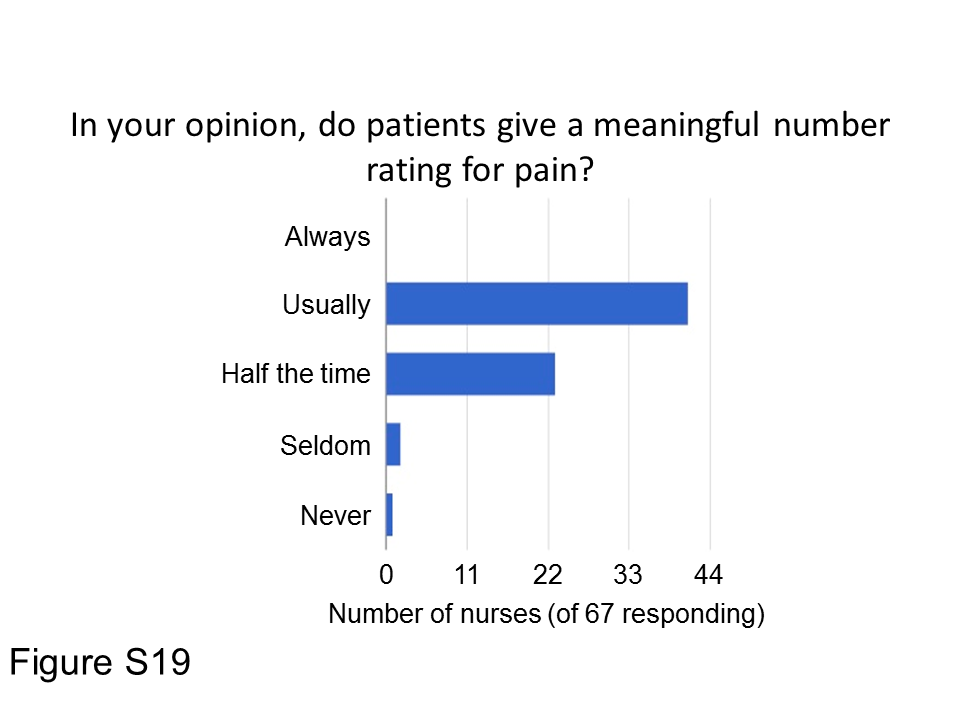

Supplement: Additional file 22: Figure S19. — In your opinion, do patients give a meaningful number rating for pain? Nurses’ perception of patient comprehension of questions. (TIF 63 kb) [file 12912_2016_196_MOESM22_ESM.tif]

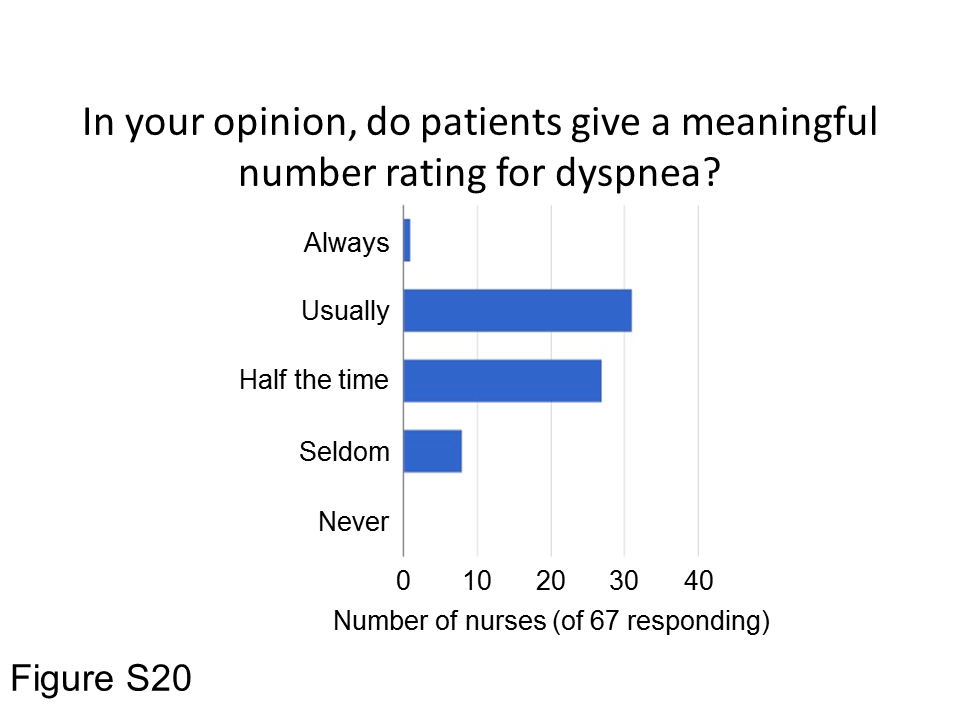

Supplement: Additional file 23: Figure S20. — In your opinion, do patients give a meaningful number rating for dyspnea? Nurses’ perception of patient comprehension of questions. (TIF 67 kb) [file 12912_2016_196_MOESM23_ESM.tif]

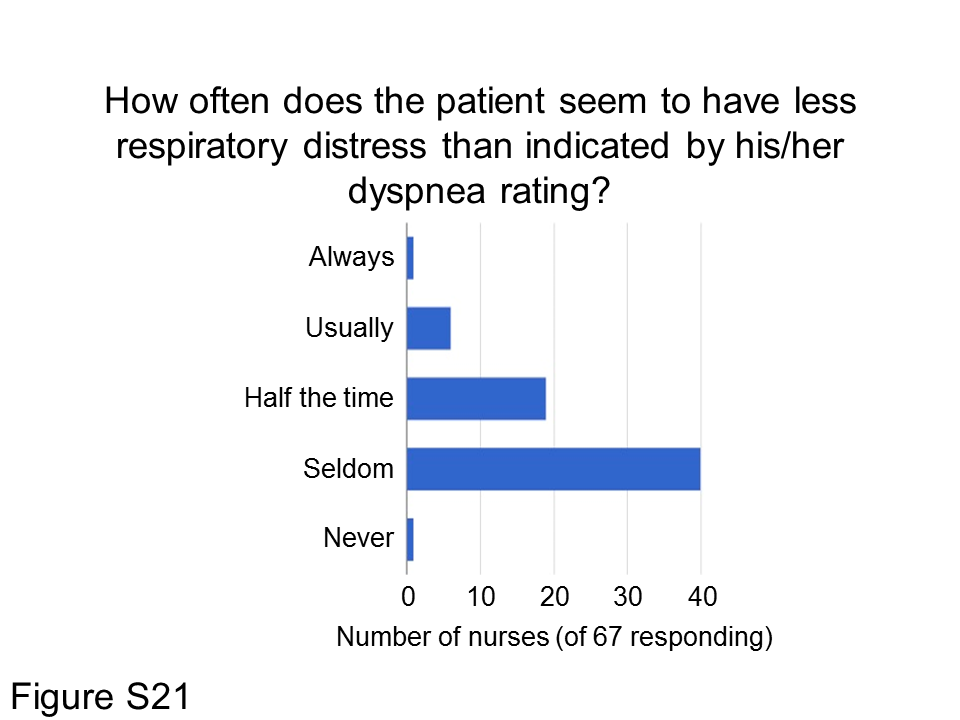

Supplement: Additional file 24: Figure S21. — How often does the patient seem to have less respiratory distress than indicated by his/her rating? Nurses’ perception of reliability of patient rating. (TIF 73 kb) [file 12912_2016_196_MOESM24_ESM.tif]

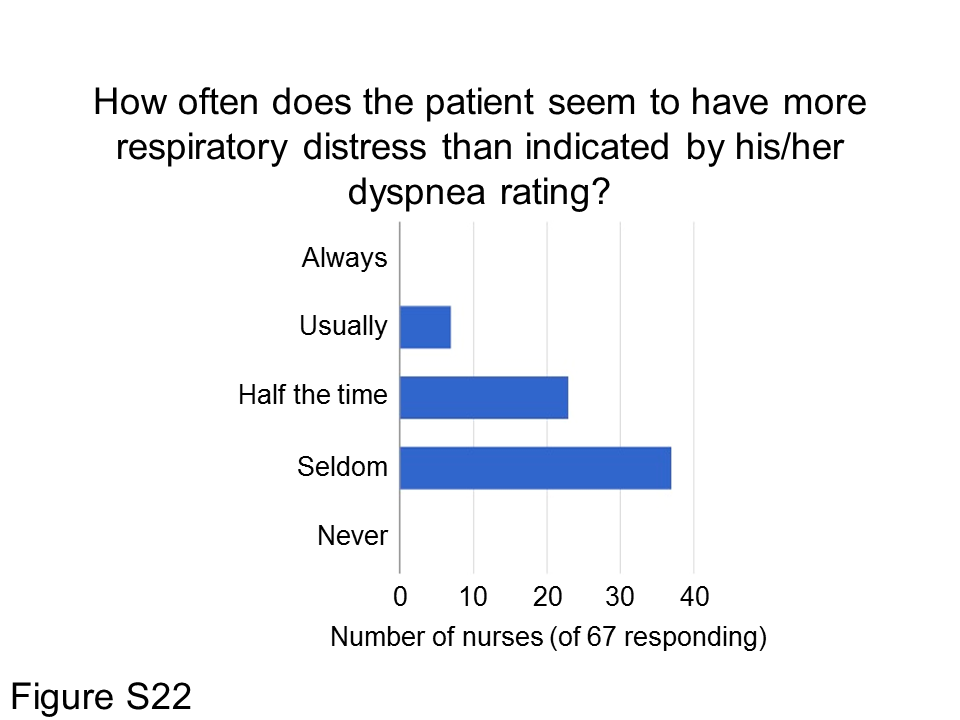

Supplement: Additional file 25: Figure S22. — How often does the patient seem to have more respiratory distress than indicated by his/her rating? Nurses’ perception of reliability of patient rating. (TIF 67 kb) [file 12912_2016_196_MOESM25_ESM.tif]

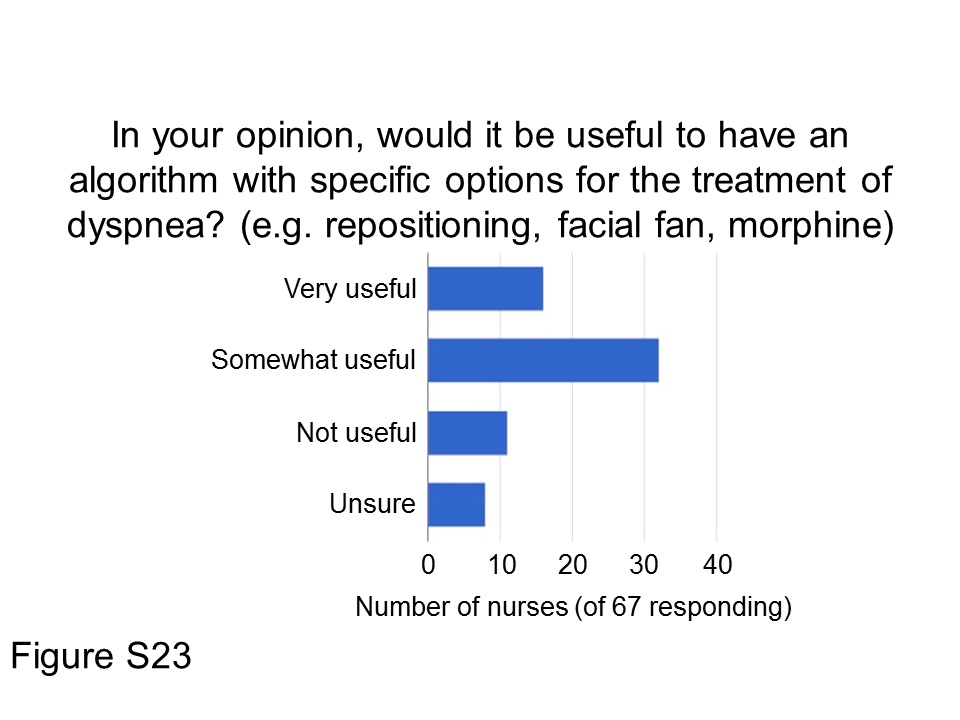

Supplement: Additional file 26: Figure S23. — In your opinion, would it be useful to have an algorithm with specific options for the treatment of dyspnea (e.g., repositioning, facial fan, morphine)? Assessment of need for support with treatment options. (TIF 80 kb) [file 12912_2016_196_MOESM26_ESM.tif]

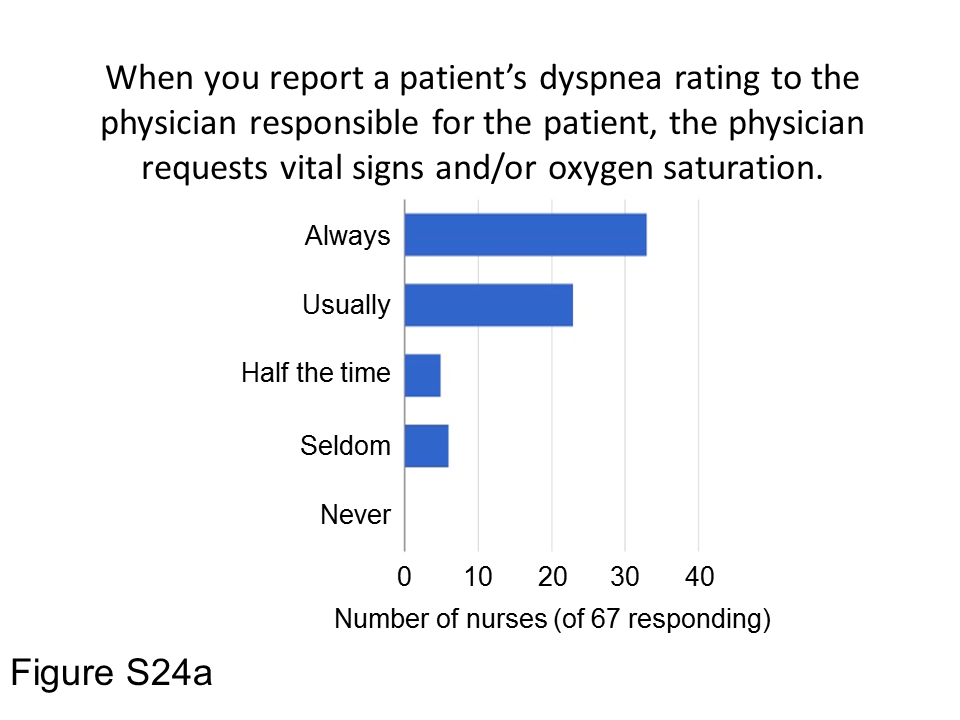

Supplement: Additional file 27: Figure S24. — a. When you report a patient’s dyspnea rating to the physician responsible for the patient, the physician requests vital signs and/or oxygen saturation. Nurses’ perception of physician response. b. When you report a patient’s dyspnea rating to the physician responsible for the patient, the physician orders laboratory or imaging studies. Nurses’ perception of physician response. c. When you report a patient’s dyspnea rating to the physician responsible for the patient, the physician orders an intervention to relieve dyspnea (pharmacologic or non-pharmacologic). Nurses’ perception of physician response. d. When you report a patient’s dyspnea rating to the physician responsible for the patient, the physician evaluates the patient. Nurses’ perception of physician response. e. When you report a patient’s dyspnea rating to the physician responsible for the patient, the physician requests nursing to reassess the patient later. Nurses’ perception of physician response. f. When you report a patient’s dyspnea rating to the physician responsible for the patient, the physician takes none of these actions. Nurses’ perception of physician response. (ZIP 467 kb) [file 12912_2016_196_MOESM27_ESM.zip › additional file 27/New S24aR2.tif]

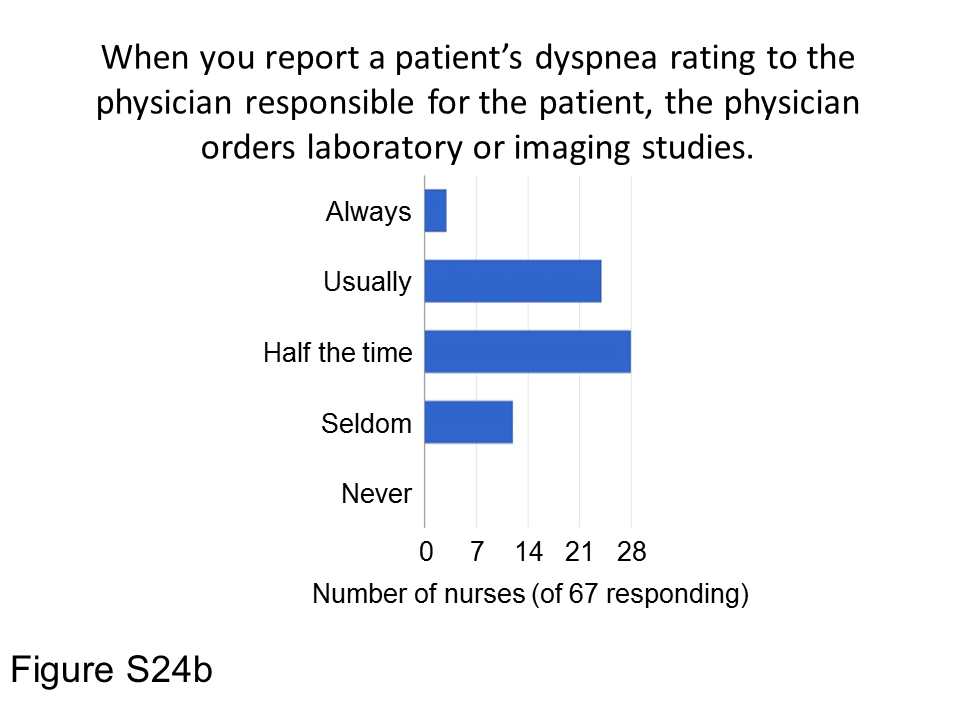

Supplement: Additional file 27: Figure S24. — a. When you report a patient’s dyspnea rating to the physician responsible for the patient, the physician requests vital signs and/or oxygen saturation. Nurses’ perception of physician response. b. When you report a patient’s dyspnea rating to the physician responsible for the patient, the physician orders laboratory or imaging studies. Nurses’ perception of physician response. c. When you report a patient’s dyspnea rating to the physician responsible for the patient, the physician orders an intervention to relieve dyspnea (pharmacologic or non-pharmacologic). Nurses’ perception of physician response. d. When you report a patient’s dyspnea rating to the physician responsible for the patient, the physician evaluates the patient. Nurses’ perception of physician response. e. When you report a patient’s dyspnea rating to the physician responsible for the patient, the physician requests nursing to reassess the patient later. Nurses’ perception of physician response. f. When you report a patient’s dyspnea rating to the physician responsible for the patient, the physician takes none of these actions. Nurses’ perception of physician response. (ZIP 467 kb) [file 12912_2016_196_MOESM27_ESM.zip › additional file 27/New S24bR2.tif]

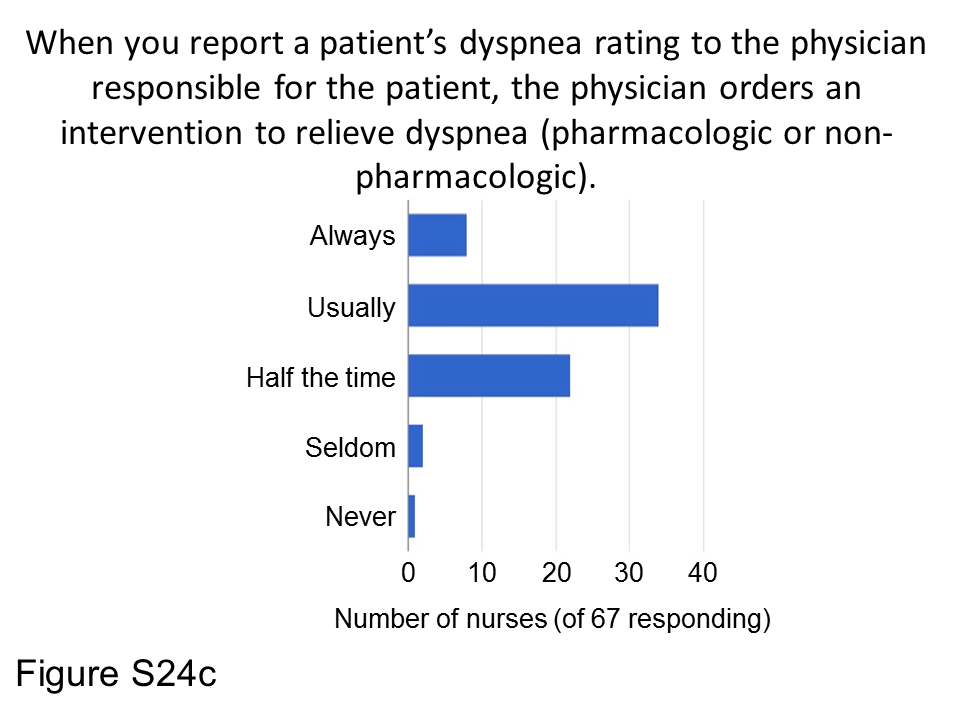

Supplement: Additional file 27: Figure S24. — a. When you report a patient’s dyspnea rating to the physician responsible for the patient, the physician requests vital signs and/or oxygen saturation. Nurses’ perception of physician response. b. When you report a patient’s dyspnea rating to the physician responsible for the patient, the physician orders laboratory or imaging studies. Nurses’ perception of physician response. c. When you report a patient’s dyspnea rating to the physician responsible for the patient, the physician orders an intervention to relieve dyspnea (pharmacologic or non-pharmacologic). Nurses’ perception of physician response. d. When you report a patient’s dyspnea rating to the physician responsible for the patient, the physician evaluates the patient. Nurses’ perception of physician response. e. When you report a patient’s dyspnea rating to the physician responsible for the patient, the physician requests nursing to reassess the patient later. Nurses’ perception of physician response. f. When you report a patient’s dyspnea rating to the physician responsible for the patient, the physician takes none of these actions. Nurses’ perception of physician response. (ZIP 467 kb) [file 12912_2016_196_MOESM27_ESM.zip › additional file 27/New S24cR2.tif]

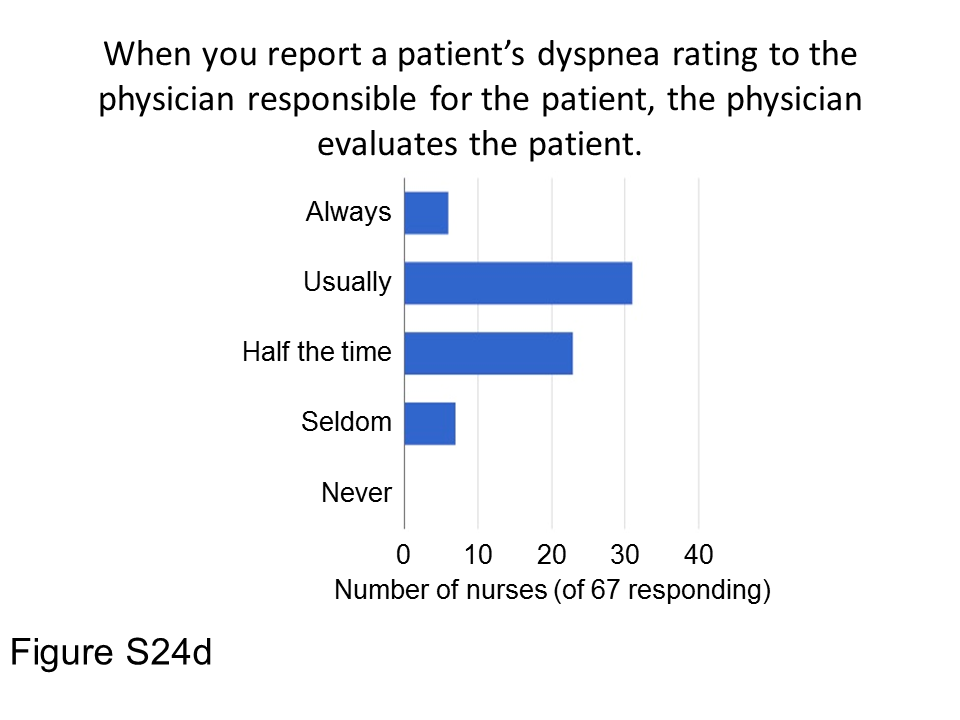

Supplement: Additional file 27: Figure S24. — a. When you report a patient’s dyspnea rating to the physician responsible for the patient, the physician requests vital signs and/or oxygen saturation. Nurses’ perception of physician response. b. When you report a patient’s dyspnea rating to the physician responsible for the patient, the physician orders laboratory or imaging studies. Nurses’ perception of physician response. c. When you report a patient’s dyspnea rating to the physician responsible for the patient, the physician orders an intervention to relieve dyspnea (pharmacologic or non-pharmacologic). Nurses’ perception of physician response. d. When you report a patient’s dyspnea rating to the physician responsible for the patient, the physician evaluates the patient. Nurses’ perception of physician response. e. When you report a patient’s dyspnea rating to the physician responsible for the patient, the physician requests nursing to reassess the patient later. Nurses’ perception of physician response. f. When you report a patient’s dyspnea rating to the physician responsible for the patient, the physician takes none of these actions. Nurses’ perception of physician response. (ZIP 467 kb) [file 12912_2016_196_MOESM27_ESM.zip › additional file 27/New S24dR2.tif]

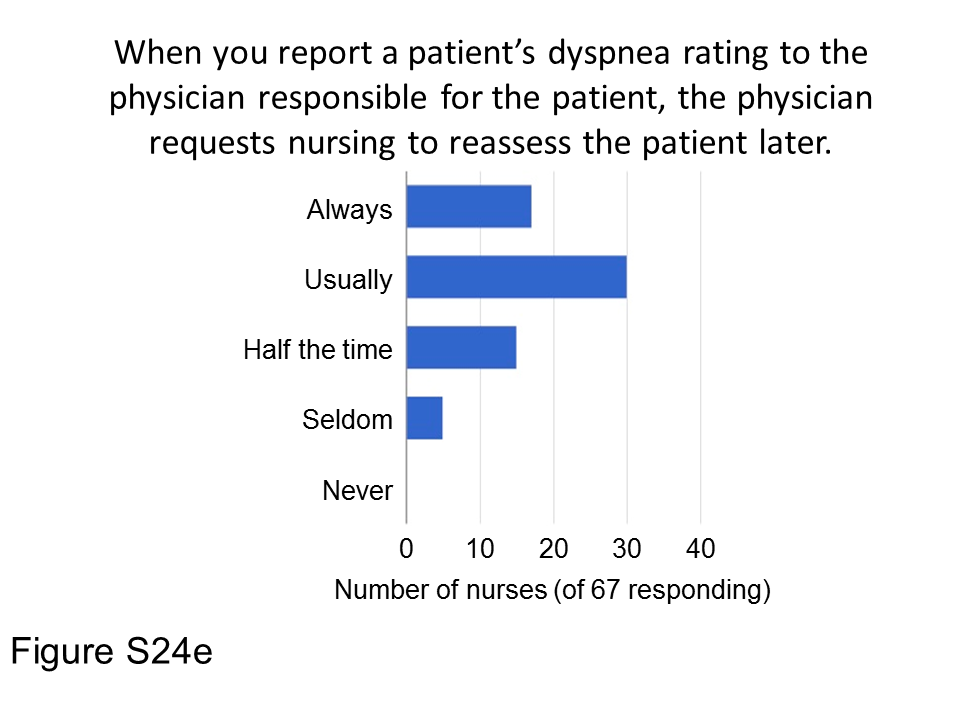

Supplement: Additional file 27: Figure S24. — a. When you report a patient’s dyspnea rating to the physician responsible for the patient, the physician requests vital signs and/or oxygen saturation. Nurses’ perception of physician response. b. When you report a patient’s dyspnea rating to the physician responsible for the patient, the physician orders laboratory or imaging studies. Nurses’ perception of physician response. c. When you report a patient’s dyspnea rating to the physician responsible for the patient, the physician orders an intervention to relieve dyspnea (pharmacologic or non-pharmacologic). Nurses’ perception of physician response. d. When you report a patient’s dyspnea rating to the physician responsible for the patient, the physician evaluates the patient. Nurses’ perception of physician response. e. When you report a patient’s dyspnea rating to the physician responsible for the patient, the physician requests nursing to reassess the patient later. Nurses’ perception of physician response. f. When you report a patient’s dyspnea rating to the physician responsible for the patient, the physician takes none of these actions. Nurses’ perception of physician response. (ZIP 467 kb) [file 12912_2016_196_MOESM27_ESM.zip › additional file 27/New S24eR2.tif]

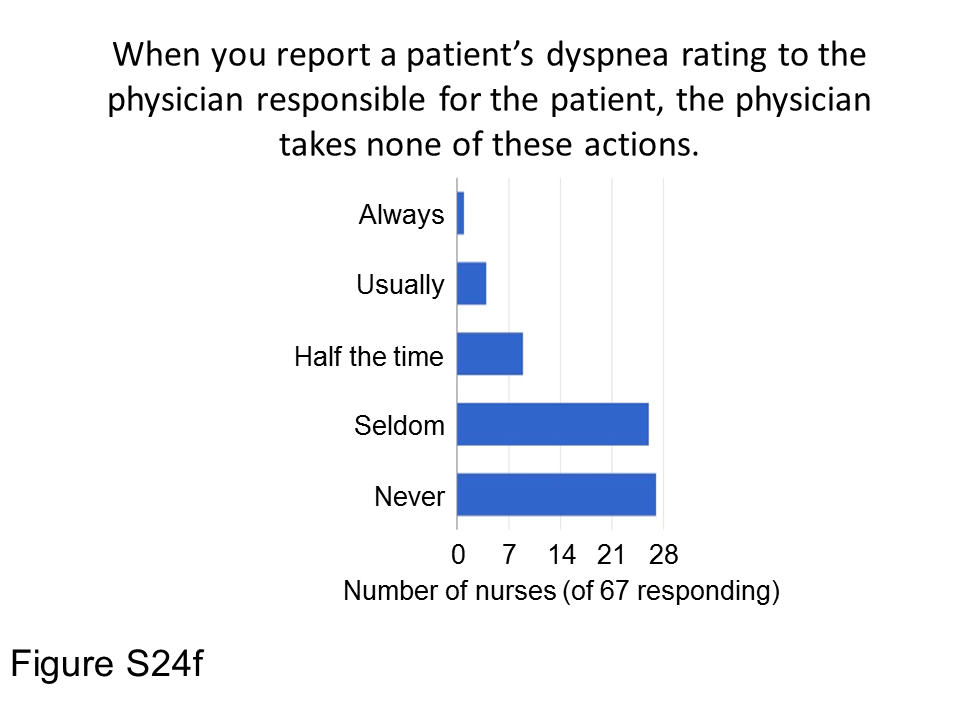

Supplement: Additional file 27: Figure S24. — a. When you report a patient’s dyspnea rating to the physician responsible for the patient, the physician requests vital signs and/or oxygen saturation. Nurses’ perception of physician response. b. When you report a patient’s dyspnea rating to the physician responsible for the patient, the physician orders laboratory or imaging studies. Nurses’ perception of physician response. c. When you report a patient’s dyspnea rating to the physician responsible for the patient, the physician orders an intervention to relieve dyspnea (pharmacologic or non-pharmacologic). Nurses’ perception of physician response. d. When you report a patient’s dyspnea rating to the physician responsible for the patient, the physician evaluates the patient. Nurses’ perception of physician response. e. When you report a patient’s dyspnea rating to the physician responsible for the patient, the physician requests nursing to reassess the patient later. Nurses’ perception of physician response. f. When you report a patient’s dyspnea rating to the physician responsible for the patient, the physician takes none of these actions. Nurses’ perception of physician response. (ZIP 467 kb) [file 12912_2016_196_MOESM27_ESM.zip › additional file 27/New S24fR2.tif]
